# Binary Pufferfish Optimization Algorithm for Combinatorial Problems

**DOI:** 10.3390/biomimetics11010010

**Published:** 2025-12-25

**Authors:** Broderick Crawford, Álex Paz, Ricardo Soto, Álvaro Peña Fritz, Gino Astorga, Felipe Cisternas-Caneo, Claudio Patricio Toledo Mac-lean, Fabián Solís-Piñones, José Lara Arce, Giovanni Giachetti

**Affiliations:** 1 Escuela de Ingeniería Informática, Pontificia Universidad Católica de Valparaíso, Avenida Brasil 2241, Valparaíso 2362807, Chileclaudio.toledo.m@mail.pucv.cl (C.P.T.M.-l.); jose.lara.a01@mail.pucv.cl (J.L.A.); 2 Escuela de Ingeniería de Construcción y Transporte, Pontificia Universidad Católica de Valparaíso, Avenida Brasil 2147, Valparaíso 2362804, Chile; 3 Escuela de Negocios Internacionales, Universidad de Valparaíso, Alcalde Prieto Nieto 452, Viña del Mar 2572048, Chile; 4 Facultad de Ingeniería, Universidad Andres Bello, Antonio Varas 880, Providencia, Santiago 7591538, Chile

**Keywords:** metaheuristic, bio-inspired algorithm, pufferfish optimization algorithm, binarization, 68T20, 68W25, 90C27, 90C59, 68Q25

## Abstract

Metaheuristics are a fundament pillar of Industry 4.0, as they allow for complex optimization problems to be solved by finding good solutions in a reasonable amount of computational time. One category of important problems in modern industry is that of binary problems, where decision variables can take values of zero or one. In this work, we propose a binary version of the Pufferfish optimization algorithm (BPOA), which was originally created to solve continuous problems. The binary mapping follows a two-step technique, first transforming using transfer functions and then discretizing using binarization rules. We study representative pairings of transfer functions and binarization rules, comparing our algorithm with Particle Swarm Optimization, Secretary Bird Optimization Algorithm, and Arithmetic Optimization Algorithm with identical computational budgets. To validate its correct functioning, we solved binary problems present in industry, such as the Set Covering Problem together with its Unicost variant, as well as the Knapsack Problem. The results we achieved with regard to these problems were promising and statistically validated. The tests performed on the executions indicate that many pair differences are not statistically significant when both methods are already close to the optimal level, and significance arises precisely where the descriptive gaps widen, underscoring that transfer–rule pairing is the main performance factor. BPOA is a competitive and flexible framework whose effectiveness is mainly governed by the discretization design.

## 1. Introduction

In today’s industry, there are significant combinatorial optimization problems such as the following: parameter optimization for cost prediction [1] (the optimization of reaction processes is crucial for the green, efficient, and sustainable development of the chemical industry [2]), optimization and robot path planning [3], and the optimization of parameters for monitoring in mining [4]. In these cases, metaheuristics are a tool for solving these problems, applying different algorithms as alternatives to exact methods. Methaheuristics are a real alternative when the problem is at a large scale, as their time is usually polynomial, achieving good solutions in a reasonable amount of time [5]. In general, metaheuristics can be applied to a wide variety of problems and have the flexibility to adapt to dynamic environments, making them suitable for different domains. These features make them highly appealing for solving real-world problems across multiple fields, including logistics [6,7], manufacturing [8,9], transportation [10,11], healthcare [12,13], and mining [14,15], among others.

However, there are another types of problems called binary problems, where the choice is to activate or deactivate a process, assign or not assign a task, or turn a machine on or off, among others. In general, these types of problems are NP-hard, which means that for large instances there are no efficient exact algorithms, meaning metaheuristics are a good-quality alternative solution with reasonable processing time. Binary metaheuristics have made contributions in a variety of industrial fields: in the logistics chain in general; in logistics in particular with regard to the allocation of routes and vehicles; in production planning when deciding which product to manufacture in different periods [16]; in the selection of financial asset portfolios under restrictions [17]; in the Crew Scheduling Problem in different areas such as maritime, land, and air where pilots, flight attendants, and drivers are assigned, taking into account a series of restrictions with the aim of minimizing costs and ensuring operations [18]; in the Workflow Scheduling in cloud computing data centers, with the aim of minimizing execution cost, execution time, and energy consumption [19]; in the security audit trail analysis problem where metaheuristics are used to improve the quality of intrusion detection [20]; in [21], a Z-shaped transfer function was used to solve the Knapsack Problem present in the industry; in [22], where a clustering-based binarization mechanism is explored to solve the Set Covering Problem, which allows modeling real-world problems where the goal is to minimize costs and maximize coverage; and in [23], where a robust Crow Search Algorithm (CSA) is proposed to solve both the optimal location of PMUs and the optimal static estimation for the entire electrical power system.

Exact algorithms (Branch and Bound or formulations based on mixed integer programming) can guarantee global optimality when the gap is zero, indicating that there is no better solution. However, these methods can have exponential behavior in the worst case for NP-complete problems, allowing metaheuristics to be an alternative solution at a lower computational cost, using exact solutions as a reference when they are available.

Within this landscape, the 0–1 Knapsack Problem (KP), the Set Covering Problem (SCP), and its unicost variant (USCP) are canonical NP-hard problems and are present in various industries [24]; notably, KP and SCP (and thus USCP) are among the 21 problems mentioned by Karp [25], and widely used as benchmarks due to their prevalence in real decision-making settings (KP) in resource selection under capacity constraints, and (SCP/USCP) in coverage- and facility-type decisions.

The use of metaheuristics to solve the KP and SCP includes algorithms such as Genetic Algorithms [26], Particle Swarm Optimization [27], the Secretary Bird Optimization Algorithm [28], the Whale Optimization Algorithm [29], the Grey Wolf Optimizer [30], the Arithmetic Optimization Algorithm [31], and the Fuzzy Hunter Optimizer [32], among others. In particular, binarization techniques have been explored to adapt metaheuristics designed for continuous optimization problems to the binary domain.

The presence of binary optimization problems in industry [33] and the use of metaheuristics leads us to take two practical approaches: on the one hand, it leads us to use algorithms created to work natively on these problems and, on the other hand, to use algorithms that were originally created to work in continuous spaces and to adapt them to work in binary spaces. Our work focuses on the second alternative, as we aim to offer a wider range of solutions for algorithms that were originally built for continuous spaces, allowing them to work in discrete environments. This is justified in order not to waste the maturity acquired by operators, such as the balance between exploration and exploitation, where POA achieves an effective balance [34,35].

The Pufferfish Optimization Algorithm (POA) is a recent bio-inspired metaheuristic that models exploration and exploitation via predator–prey dynamics and a defensive mechanism, showing competitive performance on continuous optimization tasks [36]. To apply POA (and, more generally, continuous metaheuristics) in binary combinatorial spaces such as KP and SCP, a binarization stage is required. A widely adopted approach is the two-step scheme: (i) a transfer function maps the continuous search outputs into [0,1], and (ii) a binarization rule discretizes them into {0,1} [37,38]. Transfer functions are commonly grouped into two families with distinct behaviors: S-shaped (probabilistic switching) and V-shaped (bit-flip likelihood tied to movement magnitude) [37,38]. When coupled with standard discretization rules (e.g., probabilistic STD and elitist ELIT), these functions induce distinct exploration–exploitation trade-offs within the binary search space. To provide context and comparison, PSO, AOA, and SBOA serve as standard baselines due to their well-documented behavior and established binary adaptations [27].

This work investigates how the transfer function family (S-shaped vs. V-shaped) affects the performance of a binary POA (BPOA) across maximization and minimization problem classes. For KP (maximization), we compare S1-STD versus V1-STD; for SCP and USCP (minimization), we contrast S3-ELIT versus V3-ELIT.

Our study provides a controlled comparison that clarifies the algorithmic implications of S- vs. V-shaped dynamics in BPOA and offers practical guidance for selecting transfer–rule pairs according to problem type. Concretely, our contributions are as follows: a systematic S vs. V assessment of BPOA on KP and SCP/USCP using transfer–rule combinations grounded in the prior binarization literature [37,38], and an empirical analysis against a standard baseline (PSO, AOA, and SBOA) to contextualize the performance patterns [27].

This article is organized as follows. Section 2 presents the 0–1 Knapsack Problem and Set Covering Problem. Section 3 discusses how to resolve combinatorial problems with continuos metaheuristics. Section 4 reviews the Pufferfish Optimization Algorithm. Section 5 describes explains how and make the binary version of POA. Finally, we present our results, discussions, conclusions, and possible future lines of research in Section 6, Section 7 and Section 8.

## 2. Combinatorial Problems

In this section, we present the combinatorial problems used in our work: the 0–1 KnapSack Problem, Set Covering Problem, and Unicost Set Covering Problem. Solving these problems in the context of Industry 4.0 is very important, as they constitute a basic abstraction of problems present in the industry, such as: efficient resource allocation, the efficient planning of process activities, optimal supply chain management, the optimization of sensor placement and key actions in smart industry, and route planning to ensure customer coverage, among others. Consequently, these problems define a path to practical solutions in the new industrial revolution, the study and understanding of which facilitates real-time decision-making, a key element for competitiveness in today’s world.

The Industry 4.0, which is associated with the fourth industrial revolution, constantly challenges us to improve [39,40]. Its essence lies in automated and interconnected industrial production, where technology plays a central role. Its main objective is to create highly connected, flexible, and autonomous smart industries. Industry 4.0 aims to achieve maximum production efficiency alongside agile and flexible processes with a high capacity for adaptation, optimizing the use of resources at the lowest possible cost while maintaining quality [41]. Among its fundamental pillars are the following:

- The Internet of Things (IoT). This allows different devices such as machines, sensors, tools, and products to be connected for the purpose of obtaining real-time data [42].
- Big Data and Analytics. This allows you to store and analyze large volumes of collected data, enabling you to make preventive decisions. [43].
- Automation and Advanced Robotics. These facilitate or replace repetitive tasks performed by humans [44].
- Cloud computing. This enables cloud storage and processing, allowing fast and secure access [45].
- Cybersecurity. Given the current state of digital interconnection, it is necessary to protect data to avoid risks to the industry [46].
- Additive Manufacturing. This allows objects to be created digitally before being transferred to physical form, thereby preventing errors and losses [47].
- Augmented Reality. Allows you to simulate scenarios for staff training or product improvements under special conditions [48].

Optimization is fundamental in Industry 4.0, as the data generated allows for the improvement of different industrial processes, reducing waste and improving quality and efficiency [40,49,50]. In this sense, metaheuristics are a key tool for tackling real industrial problems, by finding efficient solutions in a timely manner [51,52].

### 2.1. 0–1 Knapsack Problem

The Knapsack Problem (KP) is a classic binary combinatorial optimization problem that falls into the category of NP-hard problems. Its primary objective is to select a subset of items that maximizes the total value without exceeding a predefined maximum capacity.

#### 2.1.1. Formal Mathematical Formulation

Formally, let $O=\{o_{1},o_{2},\ldots ,o_{n}\}$ be a set of *n* available items, where each item $o_{i}$ has an associated value $v_{i}$ and weight $w_{i}$. Additionally, let *W* be the maximum weight capacity that the knapsack can hold. The problem consists of identifying an optimal subset of items that maximizes the total value without exceeding the maximum capacity *W*.

To model and computationally solve this problem, the formal set-based definition translates into a mathematical formulation using binary variables. We introduce the binary variable $x_{i}$, which takes the value of one if the item $o_{i}$ is selected to be included in the knapsack, and the value of zero otherwise.

Thus, the primary objective is to maximize the total value of the selected items, summing only the values of the included items, as indicated by Equation (1).

(1) $$\max\sum_{i=1}^{n}v_{i}x_{i}$$

The main constraint of the problem is the weight constraint, which ensures that the sum of the weights of the selected items does not exceed the maximum capacity *W*. This is represented by the following equation:

(2) $$\sum_{i=1}^{n}w_{i}x_{i}\leq W$$

Additionally, it must be ensured that the decision variable is binary:

(3) $$x_{i}\in \left\{0,1\right\},\;i=1,\ldots ,n$$

#### 2.1.2. KP Practical Example

Suppose a process plant has a planned shutdown window of W=10 h to service a bottleneck machine. Each maintenance task $o_{i}$ requires a certain duration (hours) and yields an expected benefit (e.g., avoided downtime or cost savings). The decision is binary: execute the task ($x_{i}=1$) or skip it ($x_{i}=0$). The goal is to maximize the total benefit without exceeding *W*.

#### 2.1.3. How the Optimal Set Is Obtained

To compute an optimal selection under the shutdown limit W=10 h, we can use the classical dynamic programming (DP) scheme for the 0–1 Knapsack. Let $w_{i}$ and $v_{i}$ denote the duration (hours) and the benefit of task *i*, respectively. Define

DP[i,t]=max{totalvalueusingthefirstitaskswithinthours},

with base cases DP[0,t]=0 for all t∈[0,W]. The recurrence is

$$\mathrm{DP}\left[i,t\right]\;=\;\left\{\begin{array}{cc} \max\left(\mathrm{DP}\left[i-1,t\right],\;v_{i}+\mathrm{DP}\left[i-1,\;t-w_{i}\right]\right), & \mathrm{if}\;w_{i}\leq t,\\ \mathrm{DP}[i-1,t], & \mathrm{if}\;w_{i}>t\;. \end{array}\right.$$

After filling the table up to DP[n,W], the selected tasks are recovered by backtracking; if DP[i,t]≠DP[i−1,t], then task *i* is chosen ($x_{i}=1$) and we set $t\leftarrow t-w_{i}$; otherwise task *i* is skipped ($x_{i}=0$). This yields an optimal set in O(nW) time.

##### Multiple Optimal Solutions in This Instance

For the maintenance data in Table 1 (Section 2), the optimal value is DP[6,10]=160. There are several optimal sets achieving this value, such as the following examples:

- Backup motor replacement (5 h, 80);
- Shaft alignment (3 h, 50);
- Advanced lubrication (2 h, 30);
- Backup motor replacement (5 h, 80);
- Belt replacement (4 h, 65);
- PLC update (1 h, 15);
- Shaft alignment (3 h, 50);
- Belt replacement (4 h, 65);
- Advanced lubrication (2 h, 30);
- PLC update (1 h, 15).

In the example shown previously we reported one of these optimal sets.

#### 2.1.4. Sanity Check

To make the optimum “visible” at a glance, Table 2 lists the top feasible combinations (by value) not exceeding W=10 h. No feasible combination improves upon value 160.

Codes:

- SA = Shaft alignment;
- BR = Belt replacement;
- AL = Advanced lubrication;
- SC = Sensor calibration;
- PLC = PLC software update;
- BMR = Backup motor replacement.

This subsection clarifies both how the optimal solution is computed (DP with backtracking) and what it looks like (there can be multiple optimal sets with the same maximum value).

### 2.2. Set Covering Problem

The Set Covering Problem (SCP) is a classical NP-hard combinatorial optimization problem. Given a universe of requirements and a collection of candidate subsets (each with an associated cost), the goal is to select a minimum-cost family of subsets that collectively covers every requirement at least once.

#### 2.2.1. Formal Mathematical Formulation

Let $U=\{u_{1},\ldots ,u_{m}\}$ be the set of requirements and $S=\{S_{1},\ldots ,S_{n}\}$ be a family of subsets $S_{j}\subseteq U$, with cost $c_{j}\geq 0$. A binary matrix $A\in {\left\{0,1\right\}}^{m\times n}$ encodes coverage, where $a_{ij}=1$ if $u_{i}\in S_{j}$ and $a_{ij}=0$ otherwise. Using binary decision variables $x_{j}\in \left\{0,1\right\}$ to indicate whether subset $S_{j}$ is chosen, the SCP can be stated as follows:

(4) $$\min\sum_{j=1}^{n}c_{j}\;x_{j}\;s.t.\;\sum_{j=1}^{n}a_{ij}\;x_{j}\;\geq \;1\;\;\;\forall i=1,\ldots ,m,\;x_{j}\in \left\{0,1\right\}\;\;\forall j.$$

#### 2.2.2. SCP Practical Example

Consider a pipeline network divided into five critical inspection zones, $U=\{Z_{1},\ldots ,Z_{5}\}$. The maintenance team has predefined mobile inspection routes $R_{1},\ldots ,R_{6}$; each route covers a subset of zones and has an execution time (cost). The objective is to choose the set of routes that covers all zones in the least total time as shown in Table 3.

##### Routes and Costs

We denote the cost in hours in parentheses:

$$R_{1}\;\left(4\;h\right),\;R_{2}\;\left(4\;h\right),\;R_{3}\;\left(5\;h\right),\;R_{4}\;\left(3\;h\right),\;R_{5}\;\left(3\;h\right),\;R_{6}\;\left(2\;h\right).$$

#### 2.2.3. Instance Formulation

Let $x_{j}=1$ if route $R_{j}$ is selected, and 0 otherwise. The model becomes

$$\min\;4x_{1}+4x_{2}+5x_{3}+3x_{4}+3x_{5}+2x_{6}$$

$$s.t.\;\;\begin{array}{cccc} x_{1}+x_{3}+x_{5} & \geq 1 & & \left(\mathrm{cover}\;Z_{1}\right)\\ x_{1}+x_{2} & \geq 1 & & \left(\mathrm{cover}\;Z_{2}\right)\\ x_{2}+x_{3} & \geq 1 & & \left(\mathrm{cover}\;Z_{3}\right)\\ x_{2}+x_{4}+x_{5} & \geq 1 & & \left(\mathrm{cover}\;Z_{4}\right)\\ x_{3}+x_{4}+x_{6} & \geq 1 & & \left(\mathrm{cover}\;Z_{5}\right)\\ x_{j} & \in \{0,1\} & & j=1,\ldots ,6\;. \end{array}$$

#### 2.2.4. Optimal Solution

One optimal solution is to select routes $\left\{R_{2},R_{3}\right\}$ with total cost 4+5=9 h. These two routes jointly cover all zones:

$$R_{2}:\;\left\{Z_{2},Z_{3},Z_{4}\right\},\;R_{3}:\;\left\{Z_{1},Z_{3},Z_{5}\right\}\;\;\Rightarrow \;\;\left\{Z_{1},\ldots ,Z_{5}\right\}\;\mathrm{covered}.$$

No single route covers all zones, and any selection of two routes with total cost <9 is infeasible (for completeness, another minimum-cost solution is $\left\{R_{2},R_{5},R_{6}\right\}$ with the same cost 4+3+2=9 h).

### 2.3. Unicost Set Covering Problem

The unicost variant of the Set Covering Problem (SCP) assumes identical column costs (i.e., $c_{j}=1$ for all j∈J). Consequently, the objective becomes selecting the fewest columns such that every row is covered at least once. This specialization emphasizes the structural aspect of coverage, abstracting away heterogeneous cost effects.

Mathematically, the model is as follows:

(5) $$\mathrm{Minimize}\;\sum_{j\in J}x_{j}$$

subject to

(6) $$\sum_{j\in J}a_{ij}x_{j}\geq 1\;\mathrm{for}\;\mathrm{all}\;i\in I,$$

(7) $$x_{j}\in \left\{0,1\right\}\;\mathrm{for}\;\mathrm{all}\;j\in J,$$

where $a_{ij}$ indicates whether column *j* covers row *i*, $x_{j}$ is a binary decision variable (1 if column *j* is selected; 0 otherwise), *I* is the set of rows, and *J* is the set of columns.

As with the general SCP, the unicost case is NP-hard and is widely encountered in applications such as scheduling, logistics, and resource allocation.

## 3. Continuous Metaheuristics Solving Combinatorial Problems

To deploy the Pufferfish Optimization Algorithm (POA) across multiple binary combinatorial problems, specifically the 0–1 Knapsack Problem (KP), the Set Covering Problem (SCP), and its unicost variant (USCP) the original continuous domain search dynamics must be coupled to a unified binarization layer so that POA operates in ${\left\{0,1\right\}}^{n}$ while preserving each problem’s feasibility structure (capacity in KP; coverage in SCP/USCP). As discussed in Section 1, widely used continuous metaheuristics such as the Secretary Bird Optimization Algorithm [28], Arithmetic Optimization Algorithm [31], Grey Wolf Optimizer [30], and Particle Swarm Optimization [53] follow the same adaptation path: a two-step scheme in which (i) a transfer function (e.g., S-shaped or V-shaped) maps real-valued updates to {0,1}, and (ii) a binarization rule (e.g., standard, complement, or elitist) discretizes them into 0/1 decisions. In this work, we adopt that paradigm to endow POA with a common binary search interface and apply it, unchanged, across KP, SCP, and USCP, with only the objective and constraint handling being problem specific.

### 3.1. Two-Step Technique

**Figure 1:**
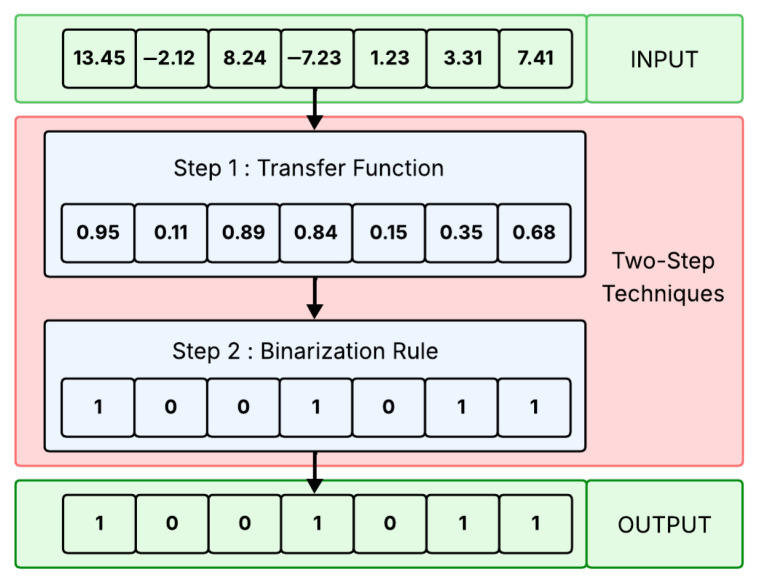
Two-Step Technique [54].

In the literature, there are different ways to binarize continuous metaheuristics [38], but the most widely used is the two-step technique [37]. as its name implies, it carries out the binarization process in two phases. In the first phase, a transfer function is applied to convert continuous solutions into the real-valued domain [0,1]. Then, in the second phase, a binarization rule is used to discretize the transferred value, thereby completing the binarization process. Figure 1 provides a general overview of this technique.

### 3.2. Transfer and Binary Functions

In the literature [38], several transfer functions have been introduced to transform continuous outputs into binary decisions. These are typically divided into two principal categories according to their profile and operational behavior: the S-shaped family and the V-shaped family. Both groups are widely recognized as standard mechanisms for translating a continuous search domain into a discrete one.

- S-Shaped Functions: Characterized by a sigmoidal curve, these functions output values in the interval [0,1], which can be interpreted as the likelihood of assigning a ‘1’ to a given component of the solution. Values near zero tend to yield probabilities close to 0.5, whereas large positive or negative inputs drive the probability towards 1 or 0, respectively. This behavior mimics a probabilistic switching process.
- V-Shaped Functions: Unlike the previous type, these functions link the probability of flipping a bit to the absolute magnitude of the continuous input, regardless of its sign. Small magnitudes lead to a low probability of change, while larger magnitudes increase that probability. This approach is conceptually related to the notion of movement intensity in swarm-based methods, where greater displacement is more likely to modify the current state of the solution.

**Figure 2:**
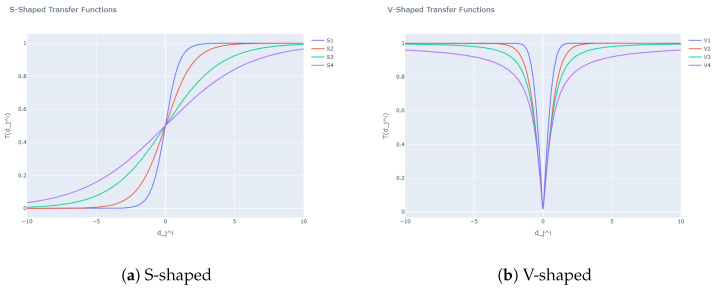
S-shaped and V-shaped transfer functions.

Table 4 and Figure 2 summarize the representative transfer functions from both families that are frequently adopted in binary optimization studies. In these expressions, $d_{j}^{i}$ denotes the continuous value at the *j*-th dimension of the *i*-th candidate, obtained after applying the perturbation defined by the continuous metaheuristic.

Additionally, in the literature [38], we can find five different binarization rules, of which we highlight the following:

- Standard (STD): If the condition is satisfied, the standard binarization rule returns the value of 1; otherwise, it returns 0. Mathematically, it is defined as follows:
  (8) $$X_{\mathrm{new}}^{j}=\left\{\begin{array}{cc} 1 & \mathrm{if}\;\mathrm{rand}\leq T(d_{i}^{j}),\\ 0 & \mathrm{else}. \end{array}\right.$$
- Elitist (ELIT): The best value is assigned if a random value is within the probability; otherwise, a zero value is assigned. Mathematically, it is defined as follows:
  (9) $$X_{\mathrm{new}}^{k}=\left\{\begin{array}{cc} X_{\mathrm{Best}}^{k} & \mathrm{if}\;\mathrm{rand}<T(d_{i}^{k}),\\ 0 & \mathrm{else}. \end{array}\right.$$

## 4. Pufferfish Optimization Algorithm

The Pufferfish Optimization Algorithm (POA) is a bio-inspired metaheuristic proposed in 2024 by Al-Baik et al. [36], based on the natural defensive behavior of the pufferfish against its predators. This algorithm was originally designed to solve continuous optimization problems, using two main phases: exploration (predator attack) and exploitation (defense mechanism), aiming to efficiently find optimal solutions.

The algorithm operates in two primary phases that mimic the natural behavior of the pufferfish:

### 4.1. Exploration Phase

This phase models the predator attack on the pufferfish, driving a global exploration of the search space. The process is defined as follows:

- Candidate Prey Selection: Each member of the population acts as a predator. For each predator, the candidate prey (other pufferfish) are randomly selected among those individuals with better objective function values. The candidate prey set $CP_{i}$ for predator *i* is defined as follows:
  (10) $$CP_{i}=\left\{X_{k}:F_{k}<F_{i}\;\mathrm{and}\;k\neq i\right\},\;i=1,2,\ldots ,N\;\mathrm{and}\;k\in \left\{1,2,\ldots ,N\right\}$$
  where
  – $CP_{i}$: The set of candidate prey for the *i*-th predator;
  – $X_{k}$: The *k*-th population member, potential prey;
  – $F_{k}$: The objective function value of the *k*-th population member;
  – $F_{i}$: The objective function value of the *i*-th predator.
- Predator Movement Towards Prey: A new position for each predator is calculated in the solution space, simulating movement toward a randomly selected prey from the candidate set. This exploration strategy enables the discovery of promising regions in the search space. The movement is modeled by the following equation:
  (11) $$x_{i,j}^{P1}=x_{i,j}+r_{i,j}\cdot \left(SP_{i,j}-I_{i,j}\cdot x_{i,j}\right)$$
  where
  – $x_{i,j}^{P1}$: New position of predator *i* in dimension *j*;
  – $SP_{i,j}$: Prey randomly selected from the set $CP_{i}$;
  – $r_{i,j}$: A random number uniformly distributed in [0,1];
  – $I_{i,j}$: A value randomly chosen as one or two, introducing variability in the movement.

In the implementation of the movement equations (Equations (11) and (12)), the random numbers $r_{i,j}$ are generated from a uniform distribution U(0,1). The variability parameter *I* in Phase 1 is randomly selected from a discrete set {1,2}.

### 4.2. Exploitation Phase

This phase captures the pufferfish’s defense mechanism against its predators, supporting a refined local search around promising regions:

- Predator Escape from Inflated Pufferfish: When attacked, the pufferfish inflates into a spiny ball, causing the predator to flee. This escape movement is translated into a local search strategy that helps to refine and exploit promising regions of the search space. The movement is modeled as follows:
  (12) $$x_{i,j}^{P2}=x_{i,j}+\left(1-2\cdot r_{i,j}\right)\cdot \frac{ub_{j}-lb_{j}}{t}$$
  where
  – $x_{i,j}^{P2}$: New position of predator *i* in dimension *j*;
  – $ub_{j}$, $lb_{j}$: Upper and lower bounds for dimension *j*;
  – *t*: Current iteration counter;
  – $r_{i,j}$: A random number uniformly distributed in [0,1].

### 4.3. Solution Selection

A key aspect of POA is its greedy selection mechanism, similar to those used by other metaheuristics, which ensures continuous improvement or at least the preservation of solutions’ quality.

The quality of each solution is evaluated using the objective function *F*. After generating a candidate solution (in either the exploration or exploitation phase), the algorithm compares the fitness value $F(X_{i}^{\mathrm{new}})$ of the new position $X_{i}^{\mathrm{new}}$ with the current value $F(X_{i})$.

The new solution is accepted only if it offers a better fitness value (i.e., a lower cost in a minimization problem). Otherwise, the current solution is retained. Formally, this is defined with the following update equation:

(13) $$X_{i}=\left\{\begin{array}{cc} X_{i}^{\mathrm{new}}, & \mathrm{if}\;F\left(X_{i}^{\mathrm{new}}\right)\leq F\left(X_{i}\right)\\ X_{i}, & \mathrm{otherwise} \end{array}\right.$$

where $X_{i}^{\mathrm{new}}$ represents a solution generated either by the predator movement or by the pufferfish’s defense mechanism. This update strategy ensures that the global quality of the population improves or at least remains stable in each iteration, thus guiding the search toward optimal solutions.

The pseudocode of POA is detailed in the pseudocode of Algorithm 1.
**Algorithm 1** Pseudocode of Pufferfish Optimization Algorithm**Input:** Input problem information: variables, objective function, and constraints.**Output:** Best solution1:Initialize the population randomly.2:**for** t=1→T **do**3:    **for** i=1→N **do**4:        **Exploration Phase:**5:        Determine the candidate Pufferfish set for the *i*-th POA member Equation (10).6:        Select the target pufferfish for the ith POA member at random.7:        Calculate new position of ith POA member using Equation (11)8:        Update the *i*-th Pufferfish using Update Equation (13).9:        **Exploitation Phase:**10:        Calculate new position of ith POA member using Equation (12).11:        Update the *i*-th Pufferfish using Update Equation (13).12:    **end for**13:    Output the best quasi-optimal solution obtained with the POA.14:    Save the best candidate solution so far.15:**end for**16:Output the best quasi-optimal solution obtained with the POA.

## 5. Binary Pufferfish Optimization Algorithm

As explained in Section 4, the Pufferfish Optimization Algorithm (POA) is a metaheuristic originally designed to solve continuous optimization problems. To address combinatorial problems such as KP, SCP, and USCP, it is necessary to transform the solutions into the binary domain.

In this work, following Section 3, the two-step technique is employed, which is one of the most widely used approaches for binarizing continuous metaheuristics [37,38]. In this scheme, the first step corresponds to the application of transfer functions that map continuous values into the interval [0,1]. For this purpose, S-shaped and V-shaped transfer functions are considered, as they provide a good balance between exploration and exploitation.

The second step corresponds to applying binarization rules that discretize the transferred values into zero or one. In this study, two well-known rules are considered: the standard rule (STD), which assigns a binary value based on a probabilistic threshold, and the elitist rule (ELIT), which favors the incorporation of the best solution found in the population.

By combining S-shaped and V-shaped transfer functions with the STD and ELIT binarization rules, we analyze the effect of these configurations on the performance of the algorithm. As reported in the literature [38], the choice of the transfer function and the binarization rule can significantly influence the quality of the solutions obtained.

With these configurations, the Binary Pufferfish Optimization Algorithm (BPOA) is constructed. The process begins with the initialization of binary solutions, which are updated in each iteration using Equations (10)–(13), which represent the specific movement equations of POA. After perturbation, the solutions temporarily leave the binary domain; therefore, the binarization process is applied using the selected combinations of S/V-shaped transfer functions and STD/ELIT rules. This cycle is repeated until the defined number of iterations is completed.

The pseudocode of BPOA is detailed in the pseudocode of Algorithm 2.
**Algorithm 2** Pseudocode of Binary Pufferfish Optimization Algorithm**Input:** Input problem information: variables, objective function, and constraints.**Output:** Best solution1:Initialize the population randomly.2:**for** t=1→T **do**3:    **for** i=1→N **do**4:        **Exploration Phase:**5:        Determine the candidate Pufferfish set for the *i*-th POA member Equation (10).6:        Select the target pufferfish for the *i*-th POA member at random.7:        Calculate new position of *i*-th POA member using Equation (11)8:        Update the *i*-th Pufferfish using Update Equation (13).9:        **Exploitation Phase:**10:        Calculate new position of *i*-th POA member using Equation (12).11:        Update the *i*-th Pufferfish using Update Equation (13).12:        **Binarization of population X**13:    **end for**14:    Output the best quasi-optimal solution obtained with the POA.15:    Save the best candidate solution so far.16:**end for**17:Output the best quasi-optimal solution obtained with the POA.

### 5.1. Theoretical Justification of Binarization

A fundamental criticism of many binary adaptations is the lack of “algorithmic specificity”, as binarization often acts as a generic wrapper. We contend that our selection of the binarization scheme is intrinsically linked to POA’s core exploration and exploitation mechanics.

POA’s mechanics rely on two phases with distinct movement magnitudes:

- Phase 1 (Exploration): Equation (11) generates large-magnitude movements in the search space, designed to jump to new, promising regions.
- Phase 2 (Exploitation): Equation (12) generates decreasing-magnitude movements, as the (t+1) term in the denominator shrinks the step size as iterations increase.

The suitability of the transfer function families (S-shaped vs. V-shaped) depends on their interaction with this mechanic:

- V-Shaped Functions (for SCP/USCP): These functions (e.g., V3) link the probability of flipping a bit (changing zero to one) to the magnitude of the movement. This couples perfectly with POA: in early iterations, the *large* movements from Phase 1 result in a *high* bit-flip probability, fostering exploration. In late iterations, the *small* movements from Phase 2 result in a *low* bit-flip probability, allowing the solution to stabilize (the “defense mechanism”).
- S-Shaped Functions (for KP): These functions (e.g., S1) interpret the continuous value as the probability of a bit being ’1’. This aligns conceptually with the nature of the KP, which is an “item selection” problem (deciding whether to include or not). POA’s movement generates a “desirability” vector, and the S-shaped function translates this into a selection probability.

### 5.2. Feasibility Handling

Once a continuous solution is binarized, it is likely to violate problem-specific constraints. Therefore, a problem-dependent heuristic repair mechanism is applied before its fitness is evaluated to ensure all solutions are feasible.

- For the Knapsack Problem (KP): The KP has a capacity constraint (2). For infeasible solutions (overweight), a greedy repair heuristic based on the profit-to-weight ratio (${\mathrm{profits}}_{i}/{\mathrm{weights}}_{i}$) is applied.
  1. Removal Phase: If the solution exceeds capacity, the algorithm iterates over included items in ascending order of their ratio (worst to best), removing them (setting to zero) until the solution becomes feasible.
  2. Addition Phase: Once feasible, the algorithm iterates over non-included items in descending order of their ratio (best to worst), adding them (setting to one) as long as the solution remains feasible. The final addition that causes infeasibility is reverted.
- For the Set Covering Problem (SCP/USCP): The SCP has coverage constraints (Equation (4)). For infeasible solutions (uncovered rows), a greedy trade-off heuristic is applied:
  – While the solution is infeasible, the algorithm identifies all uncovered rows. It then calculates a ratio (cost/number of newly covered rows) for every available column. The column with the most efficient (lowest) ratio is added to the solution (set to one). This process repeats until all rows are covered, ensuring feasibility.

### 5.3. Computational Complexity Analysis

To evaluate the computational overhead, we analyze the complexity per iteration. Let *N* be the population size, *n* the dimensionality (items/columns), *m* the number of constraints (rows, for SCP), and R(n,m) the cost of the repair function.

- BPOA Complexity: The total complexity per iteration is $O(N^{2}+N\cdot R\left(n,m\right))$. The $O(N^{2})$ term arises from the candidate prey search (Phase 1), and the O(N·R(n,m)) term arises from applying binarization, repair, and evaluation to each individual.
- BPSO Complexity: The complexity is O(N·R(n,m)), as it lacks the $O(N^{2})$ prey search.

The repair cost R(n,m) is problem dependent:

- For KP: The repair R(n) is O(nlogn), dominated by the ratio sorting. The total BPOA complexity is $O(N^{2}+N\cdot n\log n)$.
- For SCP/USCP: The greedy repair R(m,n) iterates $k_{rep}$ times (where $k_{rep}$ is the number of columns needed to repair). Each step involves recalculating coverage and ratios, taking O(m·n) (or O(nnz) for sparse matrices). The repair complexity is $R\left(m,n\right)=O\left(k_{rep}\cdot m\cdot n\right)$. The total BPOA complexity is $O(N^{2}+N\cdot k_{rep}\cdot m\cdot n)$.

In all cases, the polynomial overhead $O(N^{2})$ explains the slightly higher computation times for BPOA observed in Section 7.

## 6. Experimental Results

To validate our proposal, we used benchmark instances from the OR-Library [55] covering the 0–1 Knapsack Problem (KP) [56], the Set Covering Problem (SCP) [57], and the Unicost Set Covering Problem (USCP) [58]. Our approach was compared against widely used metaheuristics: the Pufferfish Optimization Algorithm (POA) [36], Secretary Bird Optimization Algorithm (SBOA) [28], Arithmetic Optimization Algorithm (AOA) [31], and Particle Swarm Optimization (PSO) [27].

### 6.1. Experimental Methodology

Before performing the main experimentation, we conducted internal tests for parameter configuration across the three problem classes (KP, SCP, and USCP). Specifically, we explored the population size in the range [10, 100] with steps of ten, and probed iteration budgets I∈{50,100,200,500}.

Table 5 shows the subset of instances used for parameter configuration. We chose these instances because they are representative of small/medium/large cases in the OR-Library portfolio. The table lists the following: the instance name, the problem type, the instance size (KP: number of items; SCP/USCP: *M* rows, *N* columns, and density of ones), and the known optimum. This experiment was conducted in a team using a Windows 10 operating system, an Intel Core i9-10900 K 3.70 GHz Processor, and 64 GB of RAM. The algorithm implementation was developed in the Python (v3.11.9) programming language, utilizing libraries such as NumPy and SciPy. All experiments were executed in serial mode. To ensure statistical independence across the 31 executions, a fixed random seed was not used; instead, each run was initialized with a different seed to capture varied stochastic behavior.

Table 6 shows the pilot evidence supporting the choice of a population size of ten and an iteration budget of 100 for the subsequent experiments. For each selected instance, we report (over 31 runs) the best, worst, and average objective value together with the average runtime in seconds and minutes. These results indicate fast convergence and diminishing returns beyond 100 iterations, while keeping the runtime reasonable and comparable across methods/configurations.

To further substantiate the choice of transfer functions and binarization rules under this budget, Table 7 compares, for each selected instance, an S-shaped versus a V-shaped configuration with their customary discretization rules. We report the average objective value, the percentage gap to the known optimum, and the average runtime (31 runs; 100 iterations). In KP, S1-STD attains the smallest gaps (quality first) while V1-STD is markedly faster (speed first). In SCP/USCP, V3-ELIT systematically improves both cost and time against S3-ELIT, especially as the instance size grows.

### 6.2. Conclusion of the Pilot

With a population of ten, 100 iterations form a robust knee point across KP, SCP, and USCP, balancing solutions’ quality and the runtime. Under this budget, S1-STD (quality first) and V1-STD (speed first) are the recommended pairings for KP, while V3-ELIT clearly dominates S3-ELIT for SCP/USCP in both cost and time, justifying their use in the main experiments.

Table 8 shows the final experimental configuration. The global settings used by all metaheuristics (All MH), the method, and problem-specific parameters are highlighted.

### 6.3. KP, SCP, and USCP Instances Resolved

Table 9 presents the KP instances, indicating for each case the instance name (Instance), the number of items (Number of Items), and the known optimal value (Optimum).

Table 10 and Table 11 show the instances used for the SCP and USCP, respectively. Each record in these tables includes the instance name (Instance), the number of constraints (M), the number of decision variables (N), the density of ones in the matrix described in Section 2 (Density (%)), and the known optimal value (Optimum).

It should be noted that the optimal values highlighted in bold and underlined do not correspond to global optima, but rather to the best results reported in the literature.

### 6.4. Results of KP, SCP, and USCP

This subsection presents the results obtained by BPOA using the parameters established in Section 6.1. The performance of BPOA is compared against three other metaheuristics: Particle Swarm Optimization (PSO), Secretary Bird Optimization Algorithm (SBOA), and Arithmetic Optimization Algorithm (AOA). Table 12 and Table 13 present the detailed results for the Knapsack Problem (KP) using the S1-STD binarization scheme. For each algorithm and instance, we report the optimal value (Opt), the best value achieved (Best), the average fitness (Avg. fitness), the worst value (Worst), the standard deviation (Std. fitness), and the relative percentage deviation (RPD) over 31 independent executions [59].

The RPD allows us to know how close a solution is to the known optimum.

(14) $$RPD=\frac{Z_{\mathrm{alg}}-Z_{\mathrm{ref}}}{Z_{\mathrm{ref}}}\times 100$$

where $Z_{\mathrm{alg}}$ is the objective function value returned by the algorithm under evaluation and $Z_{\mathrm{ref}}$ is the best known or optimal value for the problem instance.

**Figure 3:**
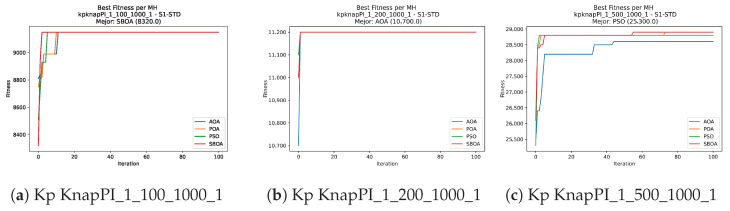
Convergence analysis of the instances Kp KnapPI_1_100_1000_1, Kp KnapPI_1_200_1000_1, and Kp KnapPI_1_500_1000_1.

**Figure 4:**
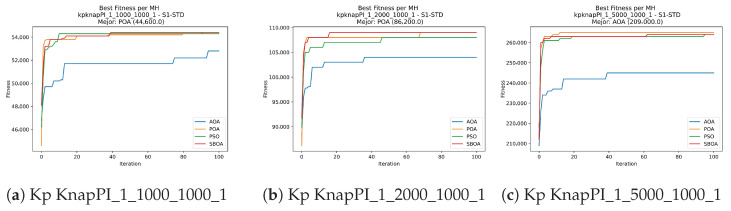
Convergence analysis of the instances Kp KnapPI_1_1000_1000_1, Kp KnapPI_1_2000_1000_1, and Kp KnapPI_1_5000_1000_1.

**Figure 5:**
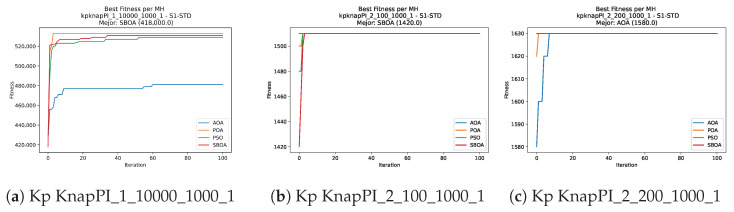
Convergence analysis of the instances Kp KnapPI_1_10000_1000_1, Kp KnapPI_2_100_1000_1, and Kp KnapPI_2_200_1000_1.

**Figure 6:**
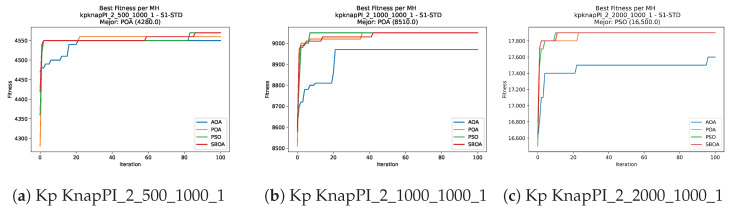
Convergence analysis of the instances Kp KnapPI_2_500_1000_1, Kp KnapPI_2_1000_1000_1, and Kp KnapPI_2_2000_1000_1.

**Figure 7:**
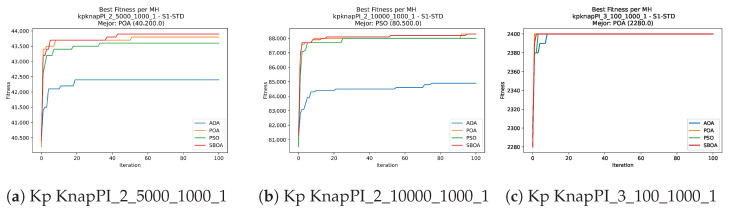
Convergence analysis of the instances Kp KnapPI_2_5000_1000_1, Kp KnapPI_2_10000_1000_1, and Kp KnapPI_3_100_1000_1.

**Figure 8:**
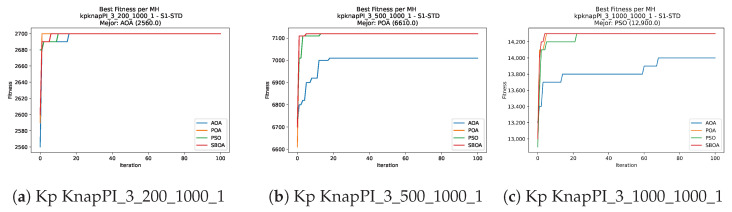
Convergence analysis of the instances Kp KnapPI_3_5000_1000_1, Kp KnapPI_2_10000_1000_1, and Kp KnapPI_3_100_1000_1.

**Figure 9:**
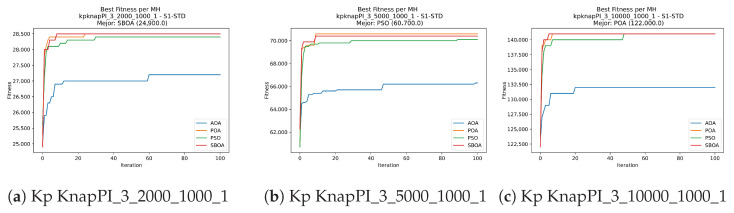
Convergence analysis of the instances Kp KnapPI_3_2000_1000_1, Kp KnapPI_3_5000_1000_1, and Kp KnapPI_3_10000_1000_1.

Convergence plots trace how the metaheuristic progressively discovers higher-quality solutions as the iteration count grows. Because practical applications demand good answers within a reasonable time, the goal is to reach strong solutions without an excessive number of iterations or computational overhead. Following Crawford et al. [60] and Lemus-Romani et al. [61], we document the search process with graphs that plot fitness against iteration. Figure 3, Figure 4, Figure 5, Figure 6, Figure 7, Figure 8 and Figure 9 present this relationship—with iterations on the x-axis and fitness on the y-axis—and they indicate the sound convergence behavior, with no evidence that the algorithm becomes trapped in a local optimum [62].

Table 14 and Table 15 summarizes runtime statistics for KP under S1–STD: minimum, maximum, average, and standard deviation (in seconds) over 31 runs for BPOA, SBOA, AOA, and PSO.

**Figure 10:**
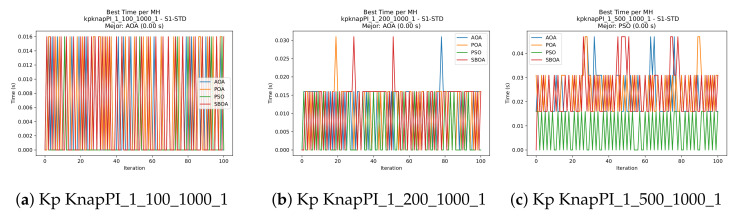
Time analysis of the instances Kp KnapPI_1_100_1000_1, Kp KnapPI_1_200_1000_1, and Kp KnapPI_1_500_1000_1.

**Figure 11:**
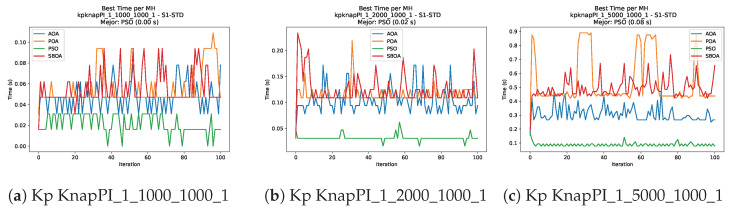
Time analysis of the instances Kp KnapPI_1_1000_1000_1, Kp KnapPI_1_2000_1000_1, and Kp KnapPI_1_5000_1000_1.

**Figure 12:**
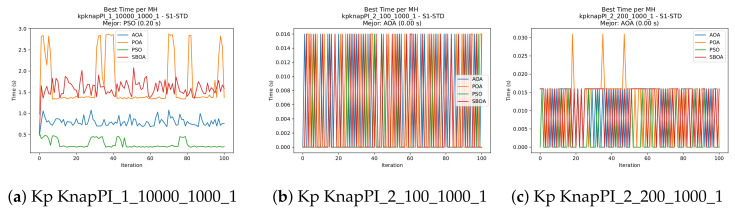
Time analysis of the instances Kp KnapPI_1_10000_1000_1, Kp KnapPI_2_100_1000_1, and Kp KnapPI_2_200_1000_1.

**Figure 13:**
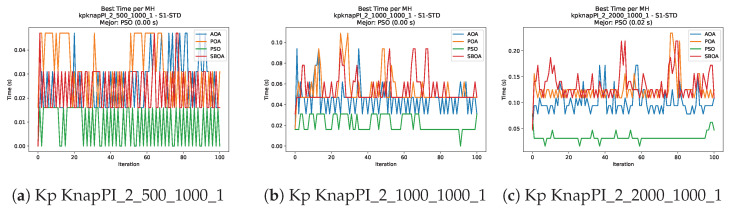
Time analysis of the instances Kp KnapPI_2_500_1000_1, Kp KnapPI_2_1000_1000_1, and Kp KnapPI_2_2000_1000_1.

**Figure 14:**
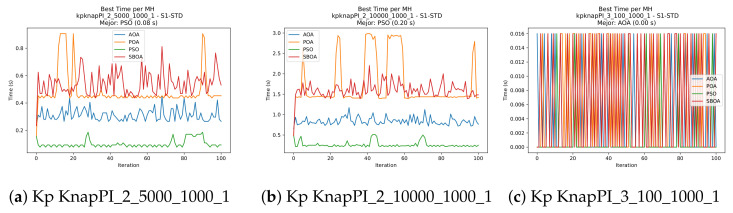
Time analysis of the instances Kp KnapPI_2_5000_1000_1, Kp KnapPI_2_10000_1000_1, and Kp KnapPI_3_100_1000_1.

**Figure 15:**
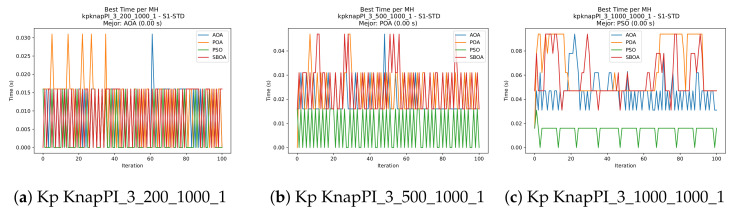
Time analysis of the instances Kp KnapPI_3_5000_1000_1, Kp KnapPI_2_10000_1000_1, and Kp KnapPI_3_100_1000_1.

**Figure 16:**
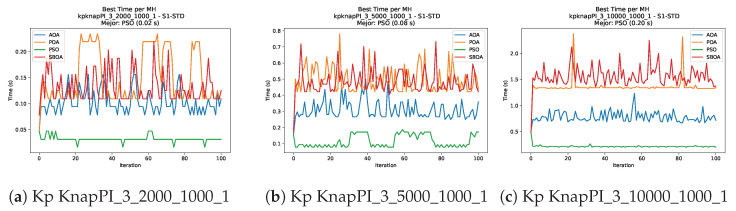
Time analysis of the instances Kp KnapPI_3_2000_1000_1, Kp KnapPI_3_5000_1000_1, and Kp KnapPI_3_10000_1000_1.

Figure 10, Figure 11, Figure 12, Figure 13, Figure 14, Figure 15 and Figure 16 show how the runtime analysis for the S1-STD configuration confirms that PSO consistently achieves the most efficient and stable execution times across all problem sizes. AOA follows as a competitive alternative, generally faster than SBOA and BPOA but with moderate variability. Conversely, BPOA and SBOA exhibit the highest computational overhead; specifically, BPOA displays noticeable spikes in large-scale instances, reflecting the intensive computational effort required to sustain its superior convergence accuracy.

Table 16 and Table 17 shows the KP solution quality when using the V1–STD binarization. The columns mirror those of Table 12 to enable a direct comparison of best/worst/mean performance and variability between BPOA, SBOA, AOA, and PSO.

**Figure 17:**
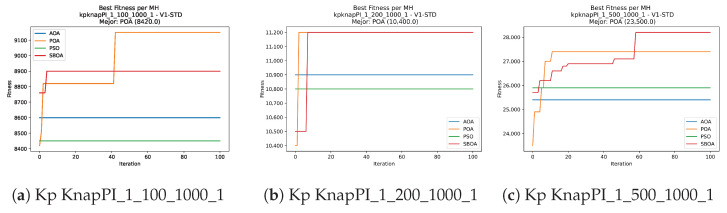
Convergence analysis of the instances Kp KnapPI_1_100_1000_1, Kp KnapPI_1_200_1000_1, and Kp KnapPI_1_500_1000_1.

**Figure 18:**
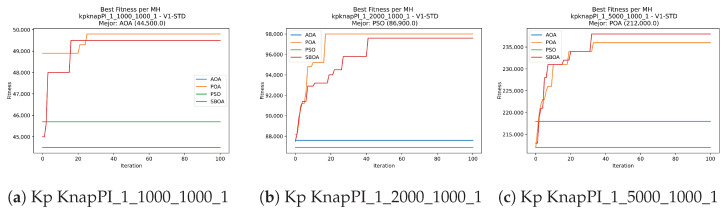
Convergence analysis of the instances Kp KnapPI_1_1000_1000_1, Kp KnapPI_1_2000_1000_1, and Kp KnapPI_1_5000_1000_1.

**Figure 19:**
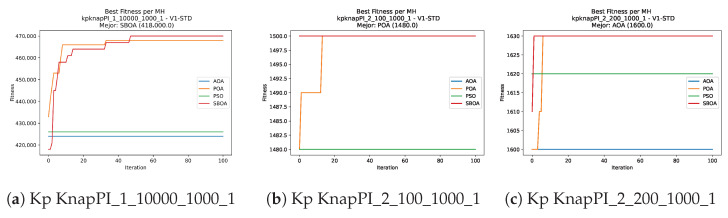
Convergence analysis of the instances Kp KnapPI_1_10000_1000_1, Kp KnapPI_2_100_1000_1, and Kp KnapPI_2_200_1000_1.

**Figure 20:**
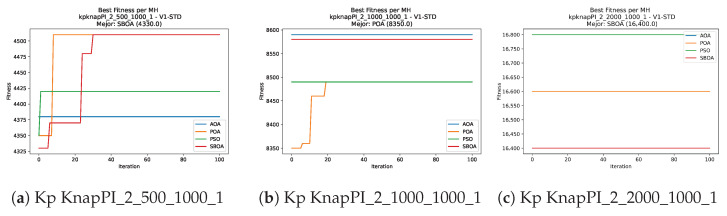
Convergence analysis of the instances Kp KnapPI_2_500_1000_1, Kp KnapPI_2_1000_1000_1, and Kp KnapPI_2_2000_1000_1.

**Figure 21:**
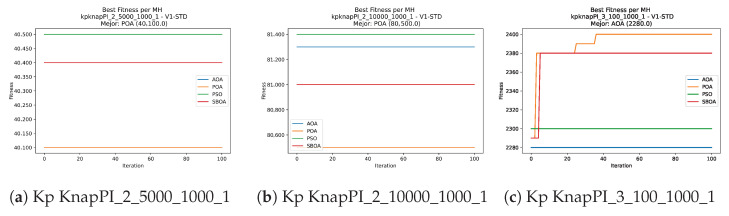
Convergence analysis of the instances Kp KnapPI_2_5000_1000_1, Kp KnapPI_2_10000_1000_1, and Kp KnapPI_3_100_1000_1.

**Figure 22:**
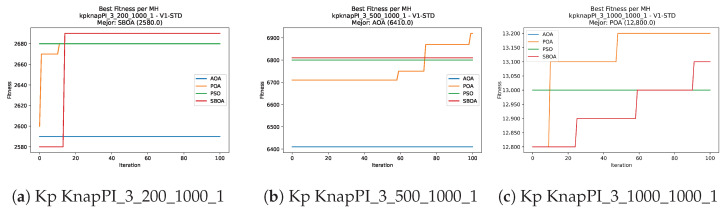
Convergence analysis of the instances Kp KnapPI_3_5000_1000_1, Kp KnapPI_2_10000_1000_1, and Kp KnapPI_3_100_1000_1.

**Figure 23:**
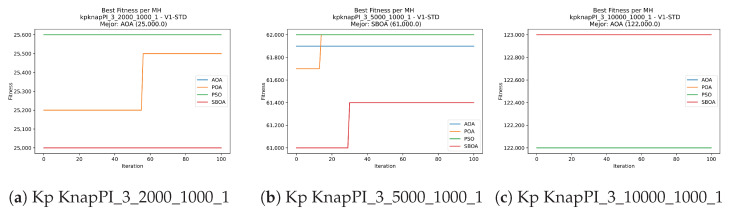
Convergence analysis of the instances Kp KnapPI_3_2000_1000_1, Kp KnapPI_3_5000_1000_1, and Kp KnapPI_3_10000_1000_1.

Figure 17, Figure 18, Figure 19, Figure 20, Figure 21, Figure 22 and Figure 23 show that BPOA and SBOA exhibit robust step-wise convergence, consistently reaching superior fitness levels compared to the stagnant performance of AOA. While PSO occasionally achieves top results in specific instances, BPOA demonstrates the most reliable trajectory, securing high-quality solutions with minimal fluctuation across the dataset.

Table 18 and Table 19 lists the runtime statistics for KP with V1–STD, allowing a time efficiency comparison against S1–STD and between the metaheuristics.

**Figure 24:**
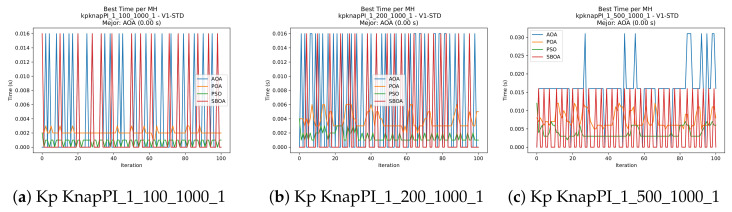
Time analysis of the instances Kp KnapPI_1_100_1000_1, Kp KnapPI_1_200_1000_1, and Kp KnapPI_1_500_1000_1.

**Figure 25:**
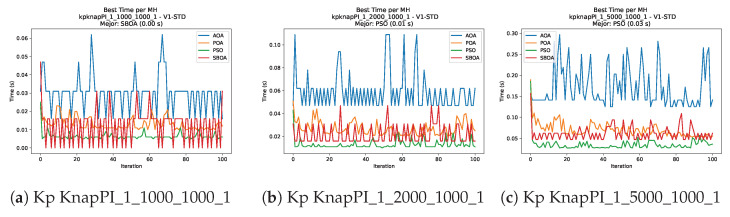
Time analysis of the instances Kp KnapPI_1_1000_1000_1, Kp KnapPI_1_2000_1000_1, and Kp KnapPI_1_5000_1000_1.

**Figure 26:**
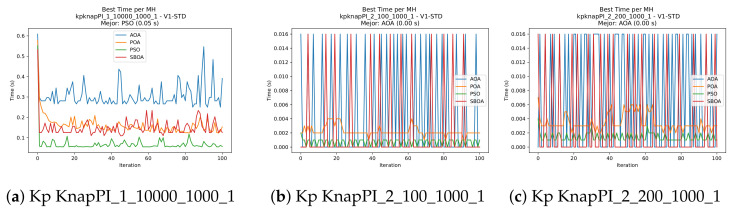
Time analysis of the instances Kp KnapPI_1_10000_1000_1, Kp KnapPI_2_100_1000_1, and Kp KnapPI_2_200_1000_1.

**Figure 27:**
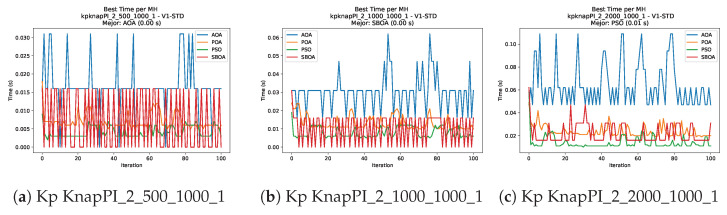
Time analysis of the instances Kp KnapPI_2_500_1000_1, Kp KnapPI_2_1000_1000_1, and Kp KnapPI_2_2000_1000_1.

**Figure 28:**
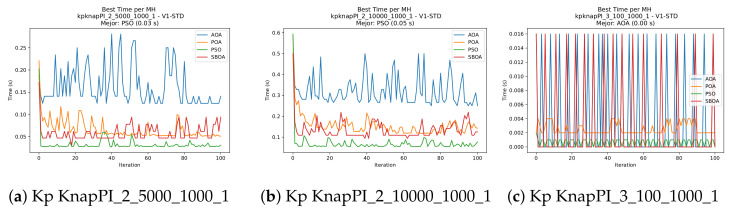
Time analysis of the instances Kp KnapPI_2_5000_1000_1, Kp KnapPI_2_10000_1000_1, and Kp KnapPI_3_100_1000_1.

**Figure 29:**
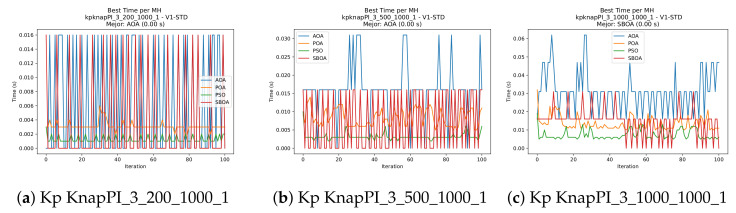
Time analysis of the instances Kp KnapPI_3_5000_1000_1, Kp KnapPI_2_10000_1000_1, and Kp KnapPI_3_100_1000_1.

**Figure 30:**
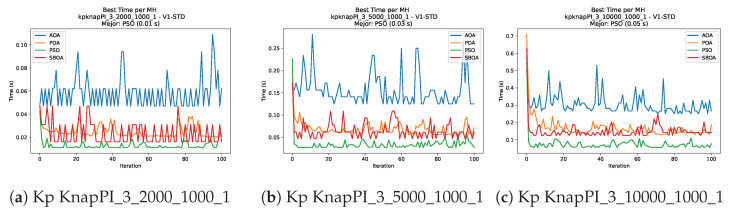
Time analysis of the instances Kp KnapPI_3_2000_1000_1, Kp KnapPI_3_5000_1000_1, and Kp KnapPI_3_10000_1000_1.

Figure 24, Figure 25, Figure 26, Figure 27, Figure 28, Figure 29 and Figure 30 show the runtime distributions, demonstrating that PSO consistently maintains the lowest and most stable execution times. In contrast, AOA exhibits significant computational instability, characterized by frequent high-magnitude spikes that increase the overall cost. BPOA and SBOA occupy an intermediate position; notably, BPOA demonstrates a steady and predictable timing behavior, effectively balancing the overhead compared to the erratic fluctuations observed in AOA.

Table 20 reports the solution costs for the Set Covering Problem (SCP) using the V3–ELIT configuration. For each instance, we include the reference optimum, best/worst value, mean over 31 runs, and standard deviation.

**Figure 31:**
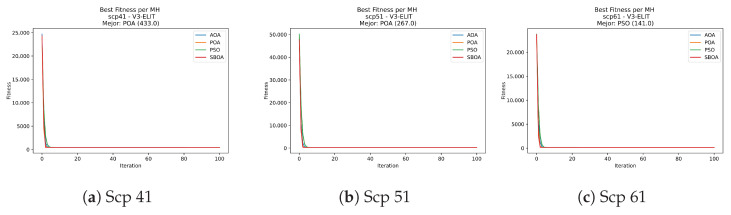
Convergence analysis of the instances Scp 41, Scp 51, and Scp 61.

**Figure 32:**
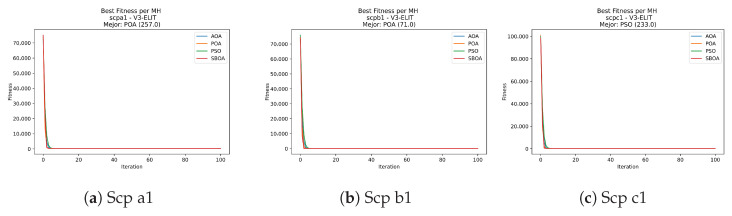
Convergence analysis of the instances Scp a1, Scp b1, and Scp c1.

**Figure 33:**
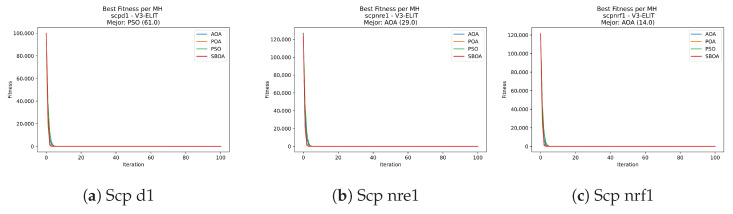
Convergence analysis of the instances Scp d1, Scp nre1, and Scp nrf1.

**Figure 34:**
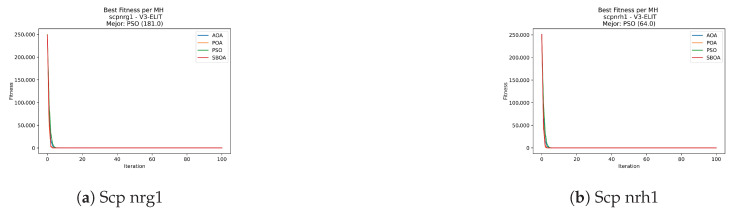
Convergence analysis of the instances Scp nrg1 and Scp nrh1.

Figure 31, Figure 32, Figure 33 and Figure 34 plot the convergence curves for SCP under V-shaped binarization, comparing POA, PSO, SBOA, and AOA. The results demonstrate that POA, PSO, and SBOA exhibit strong convergence behaviors and reach competitive final costs, whereas AOA tends to stabilize at higher values, yielding less effective solutions in most instances.

Table 21 presents the runtime statistics (min/max/avg/std, in seconds) for SCP under V3–ELIT, comparing the time performance of POA, PSO, SBOA, and AOA.

**Figure 35:**
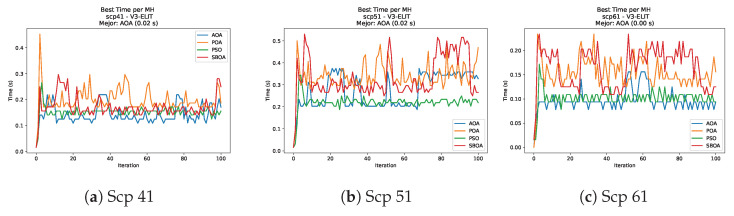
Time analysis of the instances Scp 41, Scp 51, and Scp 61.

**Figure 36:**
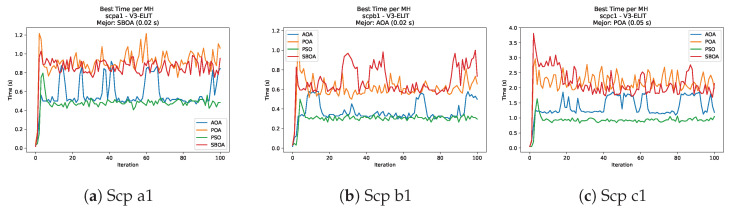
Time analysis of the instances Scp a1, Scp b1, and Scp c1.

**Figure 37:**
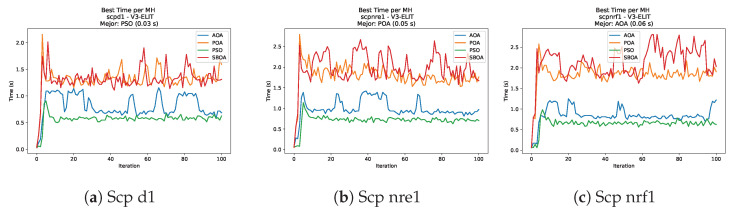
Time analysis of the instances Scp d1, Scp nre1, and Scp nrf1.

**Figure 38:**
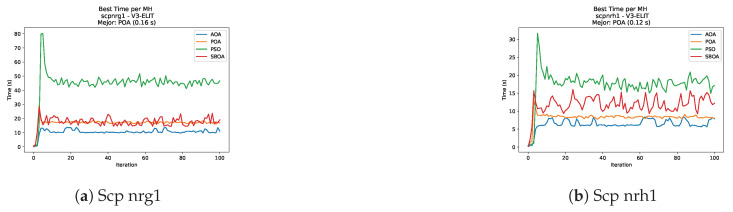
Time analysis of the instances Scp nrg1 and Scp nrh1.

Figure 35, Figure 36, Figure 37 and Figure 38 report the runtime distributions for SCP with V-shaped binarization, comparing POA, PSO, SBOA, and AOA; the results indicate that PSO is generally the most time efficient, while SBOA and AOA exhibit significantly higher computational overheads, particularly in the larger instances.

Table 22 summarizes SCP solution quality with the S3–ELIT binarization, using the same metrics as in V3–ELIT to contrast S-shaped versus V-shaped behaviors.

**Figure 39:**
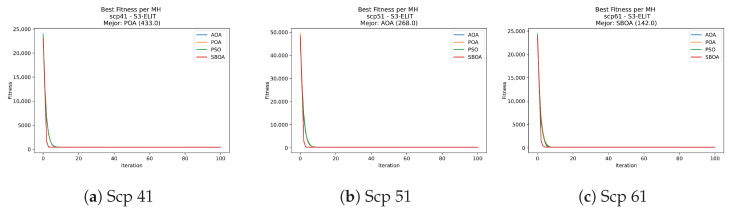
Convergence analysis of the instances Scp 41, Scp 51, and Scp 61.

**Figure 40:**
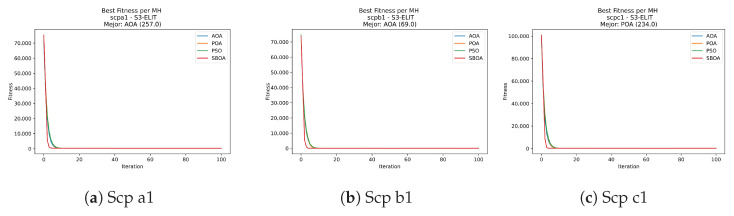
Convergence analysis of the instances Scp a1, Scp b1, and Scp c1.

**Figure 41:**
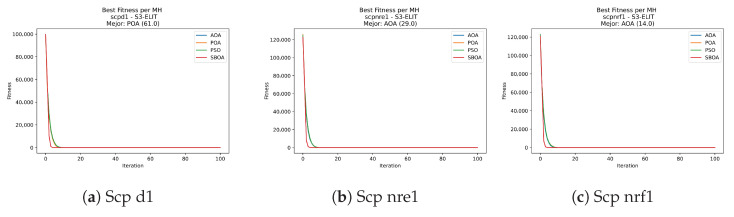
Convergence analysis of the instances Scp d1, Scp nre1, and Scp nrf1.

**Figure 42:**
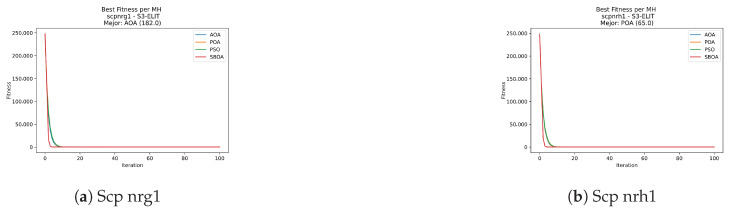
Convergence analysis of the instances Scp nrg1 and Scp nrh1.

Figure 39, Figure 40, Figure 41 and Figure 42 display convergence trajectories for SCP under S-shaped binarization, contrasting POA, PSO, SBOA, and AOA; the curves are nearly indistinguishable, with a similar early descent and virtually identical terminal costs.

Table 23 provides the SCP runtime summary with S3–ELIT, facilitating a time efficiency comparison against V3–ELIT and between the evaluated metaheuristics.

**Figure 43:**
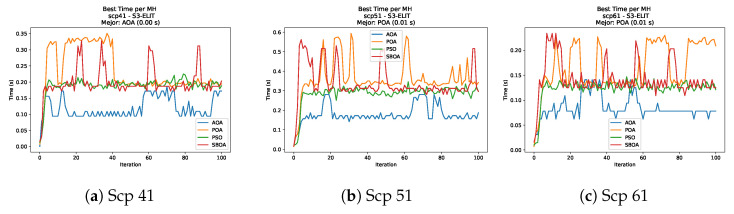
Time analysis of the instances Scp 41, Scp 51, and Scp 61.

**Figure 44:**
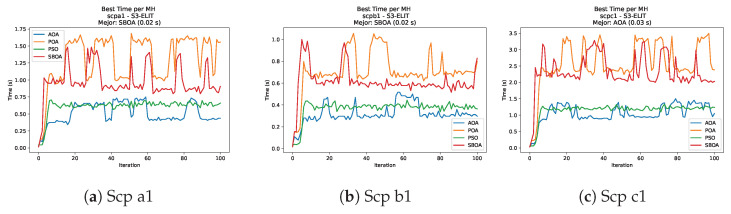
Time analysis of the instances Scp a1, Scp b1, and Scp c1.

**Figure 45:**
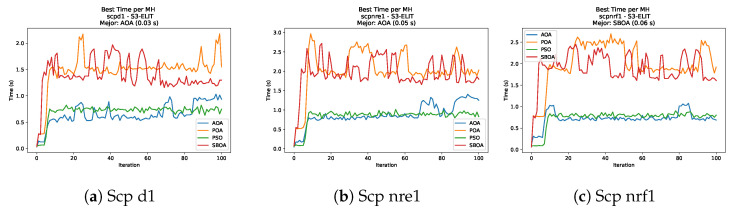
Time analysis of the instances Scp d1, Scp nre1, and Scp nrf1.

**Figure 46:**
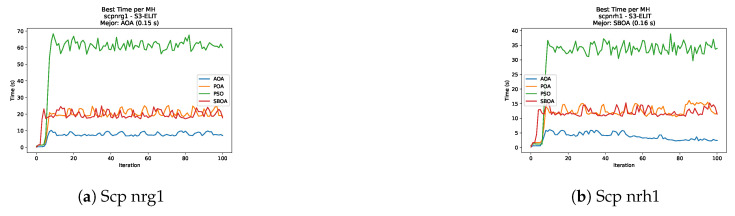
Time analysis of the instances Scp nrg1 and Scp nrh1.

Figure 43, Figure 44, Figure 45 and Figure 46 illustrate the runtime behavior per iteration for SCP instances using the S3-ELIT binarization scheme. As observed in the plots, AOA consistently exhibits the lowest computational times, demonstrating superior efficiency. PSO follows as the second fastest algorithm, maintaining a stable performance. In contrast, POA and SBOA display significantly higher runtimes and greater variability across the iterations, indicating the higher computational cost of these methods in this specific configuration.

Table 24 compiles the solution quality for the Unicost Set Covering Problem (USCP) using V3-ELIT. We report the best values, the worst values, the mean, and dispersion over 31 runs, relative to the known optimum.

**Figure 47:**
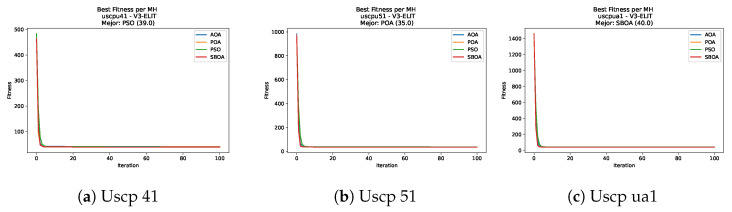
Convergence analysis of the instances Uscp 41, Uscp 51, and Uscp ua1.

**Figure 48:**
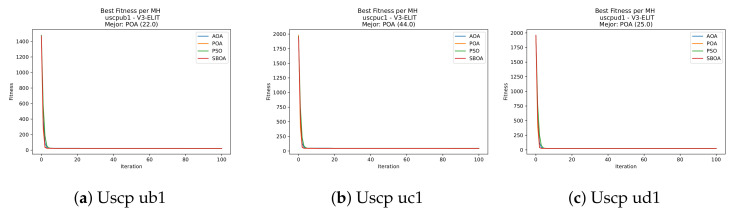
Convergence analysis of the instances Uscp ub1, Uscp uc1, and Uscp ud1.

**Figure 49:**
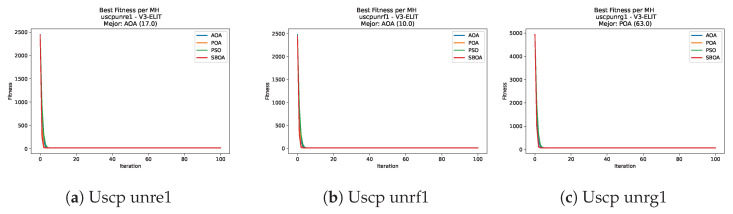
Convergence analysis of the instances Uscp unre1, Uscp unrf1, and Uscp unrg1.

**Figure 50:**
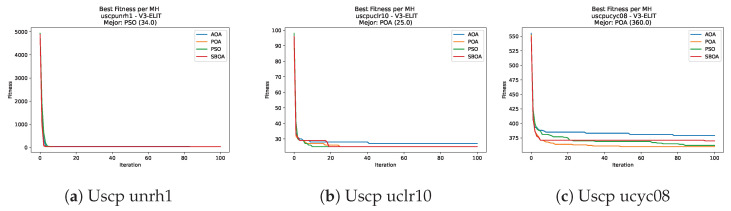
Convergence analysis of the instances Uscp unrh1, Uscp uclr10, and Uscp ucyc08.

Figure 47, Figure 48, Figure 49 and Figure 50 show the convergence curves for USCP under V-shaped binarization across multiple instances and runs. As illustrated, POA and PSO exhibit the most rapid convergence rates, settling at nearly identical optimal costs. Notably, in complex instances such as ucyc08, both algorithms clearly outperform AOA and SBOA, demonstrating their superior scalability and search capability.

Table 25 shows the runtime statistics (min, max, average, and standard deviation, in seconds) for USCP with V3–ELIT, comparing BPOA, PSO, AOA, and SBOA.

**Figure 51:**
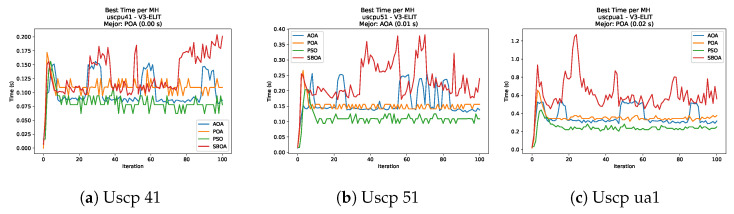
Time analysis of the instances Uscp 41, Uscp 51, and Uscp ua1.

**Figure 52:**
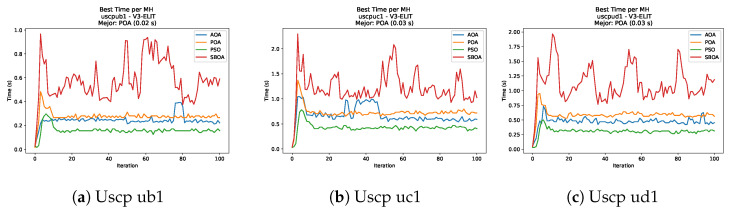
Time analysis of the instances Uscp ub1, Uscp uc1, and Uscp ud1.

**Figure 53:**
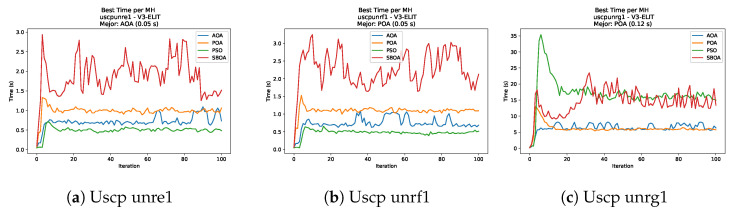
Time analysis of the instances Uscp unre1, Uscp unrf1, and Uscp unrg1.

**Figure 54:**
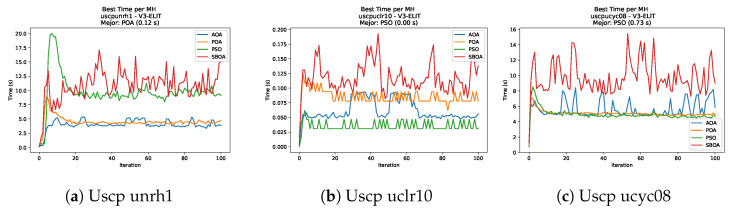
Time analysis of the instances Uscp unrh1, Uscp uclr10, and Uscp ucyc08.

Figure 51, Figure 52, Figure 53 and Figure 54 compare the runtime distributions for USCP with V-shaped binarization. PSO generally exhibits the lowest runtimes, demonstrating high speeds across most instances, although it shows instability in larger datasets (e.g., unrg1 and unrh1). Conversely, SBOA displays the highest computational overhead and variability. POA and AOA maintain stable, intermediate execution times, positioning themselves between the rapid performance of PSO and the higher costs of SBOA.

Table 26 presents USCP solution quality using S3-ELIT, with the same performance metrics as those used for V3-ELIT to highlight the impact of switching from V-shaped to S-shaped transfers.

**Figure 55:**
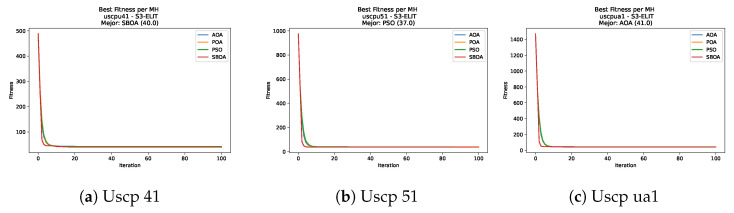
Convergence analysis of the instances Uscp 41, Uscp 51, and Uscp ua1.

**Figure 56:**
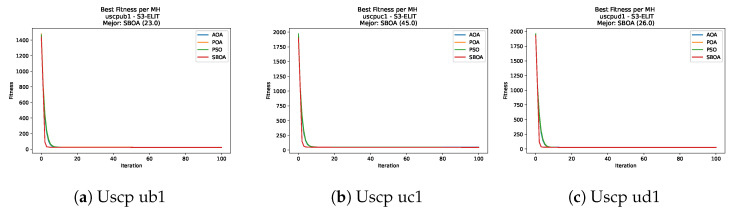
Convergence analysis of the instances Uscp ub1, Uscp uc1, and Uscp ud1.

**Figure 57:**
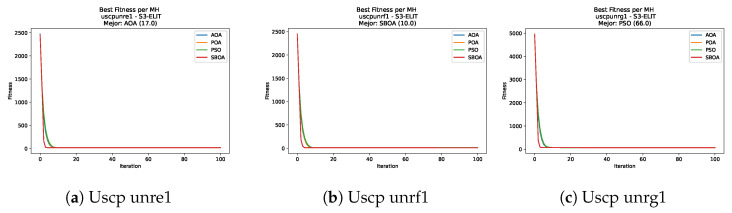
Convergence analysis of the instances Uscp unre1, Uscp unrf1, and Uscp unrg1.

**Figure 58:**
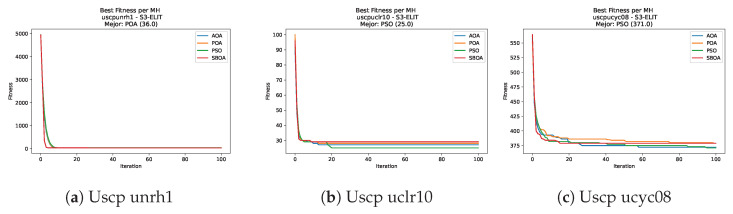
Convergence analysis of the instances Uscp unrh1, Uscp uclr10, and Uscp ucyc08.

Figure 55, Figure 56, Figure 57 and Figure 58 present the convergence profiles for USCP under S-shaped binarization; the trajectories overlap closely throughout, with matching early improvement and final costs that are effectively the same.

Table 27 reports the runtime statistics for USCP with S3–ELIT, enabling a direct time comparison with V3–ELIT and between BPOA, PSO, AOA, and SBOA.

**Figure 59:**
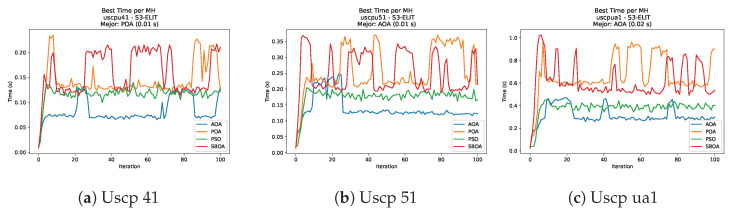
Time analysis of the instances Uscp 41, Uscp 51, and Uscp ua1.

**Figure 60:**
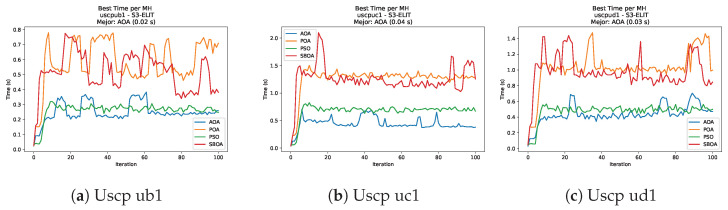
Time analysis of the instances Uscp ub1, Uscp uc1, and Uscp ud1.

**Figure 61:**
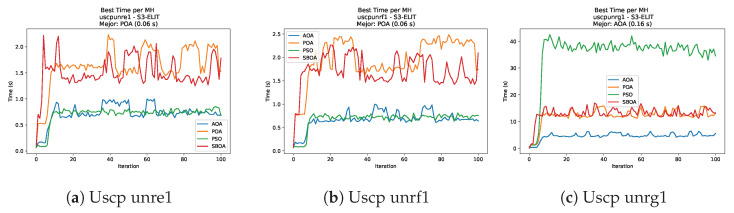
Time analysis of the instances Uscp unre1, Uscp unrf1, and Uscp unrg1.

**Figure 62:**
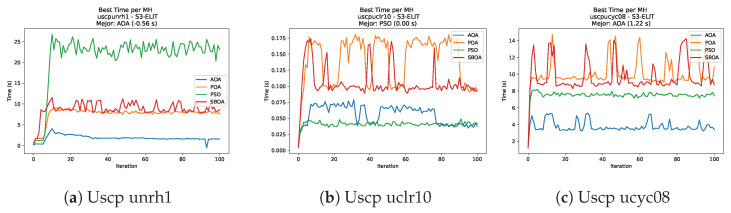
Time analysis of the instances Uscp unrh1, Uscp uclr10, and Uscp ucyc08.

Figure 59, Figure 60, Figure 61 and Figure 62 report the runtime outcomes for USCP with S-shaped binarization across instances and repetitions. AOA consistently achieves the lowest execution times, establishing itself as the most efficient method in this configuration. While PSO is competitive in smaller instances, it exhibits significant instability and high runtimes on larger datasets (e.g., unrg1 and unrh1), whereas POA maintains a stable, albeit higher, computational cost.

In the Knapsack Problem (KP), the S1-STD binarization scheme yields near-optimal and highly stable results across all algorithms, although POA and PSO maintain a slight consistency edge over AOA and SBOA; conversely, the V1-STD scheme drastically reduces the runtimes at the expense of solution quality, where AOA stands out for its speed but exhibits larger optimality gaps in large-scale instances. Regarding the Set Covering Problems (SCP and USCP), the V3-ELIT approach clearly outperforms S3-ELIT, delivering superior costs and execution times, whereas S3-ELIT demonstrates poor scalability, particularly with SBOA, which exhibits the highest computational overhead and variability. In terms of comparative performance, AOA consistently ranks as the fastest method, achieving the lowest execution times in most scenarios, although it is occasionally outperformed in terms of solution quality by POA and PSO in complex instances; PSO offers a strong balance, being rapid on smaller instances yet showing runtime instability on larger datasets, and POA demonstrates the highest stability and search capability in difficult scenarios, justifying its intermediate computational cost with high-quality solutions, while SBOA proves to be the least time efficient. Overall, the results suggest employing S1-STD for KP when quality is paramount and V1-STD for speed, and choosing V3-ELIT for SCP/USCP, selecting AOA for maximum speed or POA/PSO for an optimal trade-off between solution quality and stability.

### 6.5. Statistical Test

To statistically validate our work, we considered the POA and PSO executions for the KP, SCP, and USCP problems. Our statistical analysis is based on two stages, according to [57]. The first stage consists of determining whether the data behaves normally, for which we use the Shapiro–Wilk test [63], as shown in Table 28, Table 29, Table 30, Table 31 and Table 32, where W represents the Shapiro–Wilk statistic, whose value is between [0,1], and the *p*-value is the probability of obtaining the observed data. As can be seen, the data does not follow a normal trend. To apply the test, we use the scipy.stats function in Python.

In the second stage of our analysis, given that our data does not follow a normal distribution, we used a nonparametric test, which in this case is the Mann–Whitney test. This test is used when two samples are independent and the validity of one of them cannot be assumed. We considered the following hypotheses:

**H_0_:**  *Algorithm A* ≥ *Algorithm B,*

**H_1_:**  *Algorithm A* < *Algorithm B,*

where *Algorithm A* and *Algorithm B* represent the average value delivered by algorithms A and B. In two independent and uncorrelated data sets, we use the *p*-value to determine whether they are significantly different. In this sense, we consider that if the *p*-value is less than 0.05, the null hypothesis $H_{0}$ will be rejected, and the alternative hypothesis $H_{1}$ will be accepted. To apply the test, we use the scipy.stats function in Python. The application of the Mann–Whitney test is shown in Table 33, Table 34 and Table 35.

The Mann–Whitney test applied to the different problems with the selected pairs of techniques did not reveal statistically significant differences between the metaheuristics, as most of the *p*-values were greater than 0.05. Therefore, the null hypothesis could not be rejected.

## 7. Discussion

The empirical results validate our theoretical hypothesis (Section 5.1) regarding the synergy between POA’s mechanics and the chosen binarization scheme. For SCP/USCP, the superiority of V3–ELIT is not coincidental: POA’s Phase 1 (Exploration) generates high-magnitude movements, which the V-shaped function translates into a high bit-flip probability, fostering diversity. Conversely, Phase 2 (Exploitation) generates decreasing-magnitude movements, which the V-shaped function translates into solution stabilization, enabling convergence. S3–ELIT, lacking this coupling to movement magnitude, fails to achieve this dynamic translation.

This study investigated a binary instantiation of the Pufferfish Optimization Algorithm (BPOA) on three canonical 0–1 problems, KP, SCP, and USCP, via the two-step technique using representative S-shaped and V-shaped transfer functions combined with STD and ELIT binarization rules, benchmarking against PSO under a common budget (Table 8). The empirical evidence in Table 12, Table 13, Table 14, Table 15, Table 16, Table 17, Table 18, Table 19, Table 20, Table 21, Table 22, Table 23, Table 24, Table 25, Table 26 and Table 27 reveals several robust patterns that we summarize below.

For KP, S1–STD delivers near-optimal, low-variance solutions over a wide range of sizes, while V1–STD yields markedly shorter runtimes at the expense of larger optimality gaps and variability (cf. Table 12 and Table 14 vs. Table 16 and Table 18). This establishes a clear quality–speed trade-off: S1–STD is the quality-first option; V1–STD is a pragmatic choice when tight wall-time constraints prevail.

For coverage minimization (SCP/USCP), V3–ELIT consistently outperforms S3–ELIT in terms of solutions’ cost, robustness, and scalability, with especially clear advantages in large/dense instances, where S3–ELIT’s runtime escalates sharply (cf. Table 20, Table 21, Table 22, Table 23, Table 24, Table 25, Table 26 and Table 27). Thus, for coverage problems, the V-shaped transfer with an elitist rule provides the most favorable quality–efficiency balance.

Once an effective transfer–rule pairing is fixed, the search engine plays a secondary role: PSO is generally faster, while BPOA is occasionally more stable or slightly tighter in terms of the best/mean cost in the hardest instances, framing a time–stability trade-off. Complementarily, Wilcoxon–Mann–Whitney tests over 31 runs mostly return p>0.05 when the methods are already near optimum (e.g., KP with S1–STD; several SCP/USCP cases under V3–ELIT) and show significance precisely where descriptive gaps are large, reinforcing that discretization design is the principal performance driver and the choice between BPOA and PSO mainly modulates runtime and stability.

Mechanistically, these results align with bit-flip dynamics: S-shaped transfers with STD behave as probability thresholds that stabilize packing decisions in KP’s profit–weight landscape (hence S1–STD’s low variance), whereas V-shaped transfers increase flip probability with movement magnitude, promoting faster but less predictable exploration (V1–STD). For coverage problems, ELIT’s guidance from the incumbent best is crucial in order to propagate structural improvements under constraints, and V3’s smooth, saturating map supplies moderated but persistent flips, explaining V3–ELIT’s superior quality–efficiency trade-off. The limitations of this study include its reliance on OR-Library benchmarks and fixed budgets (Table 8), exploration of a restricted subset of transfers/rules (S1/S3 and V1/V3; STD/ELIT), standard repair/feasibility checks that could interact with pairings, and and per-instance Wilcoxon tests without multiple comparison control or effect sizes; nevertheless, across the problems and sizes, the picture remains internally consistent and practically actionable, yielding clear guidance regarding when to favor quality-first vs. speed-first settings.

## 8. Conclusions

Within the context of the importance of optimization for Industry 4.0, we presented a unified binary adaptation of POA and evaluated its effectiveness on KP, SCP, and USCP under representative transfer-–rule designs, contrasting it with PSO, AOA, and SBOA under identical computational budgets. Three main conclusions follow from the experimental evidence.

For KP, S1–STD is the most reliable configuration, delivering near-optimal, low-variance solutions across sizes while V1–STD offers a compelling speed-first regime with a predictable trade-off in optimality gap and variability. Practitioners should select between these regimes based on the wall-time constraints and acceptable deviation from the optimum.

For SCP/USCP, the discretization pairing dominates performance: V3–ELIT consistently surpasses S3–ELIT in cost, robustness, and scalability, especially in large/dense instances. This indicates that moderated flip dynamics guided by elitism are better suited to coverage minimization than S-shaped thresholds.

Between BPOA and the other algorithms, once a good combination has been chosen, the solvers achieve comparable quality. AOA is usually faster, while BPOA can be slightly more accurate or stable in difficult cases with higher computational cost; therefore, the choice of method mainly adjusts the speed–stability profile rather than determining absolute performance. Therefore, the practical recommendations are as follows: use S1–STD for KP when quality is paramount and V1–STD with strict time budgets; prefer V3–ELIT for SCP/USCP to obtain better costs with manageable execution times; select the other algorithms for performance-oriented scenarios; and select BPOA when incremental gains or stability justify additional computation. Future work will expand the design space (additional S/V transfers and rules), develop adaptive/hybrid schedules that alternate transfer and rule during execution, integrate problem-aware repairs and lightweight local improvements for coverage, adopt dynamic population/budget allocation, strengthen inference with multiple comparison procedures and effect sizes, and validate on larger and more heterogeneous industrial instances to assess generalization and cost–quality scalability.

## Figures and Tables

**Table 1:** Task data (KP maintenance example).

Task	Time (h)	Value (Benefit Units)
Shaft alignment	3	50
Belt replacement	4	65
Advanced lubrication	2	30
Sensor calibration	3	40
PLC software update	1	15
Backup motor replacement	5	80

**Table 2:** Best feasible combinations (KP maintenance example).

Tasks (Codes)	Total Time (h)	Total Value
BR + PLC + BMR	10	160
SA + AL + BMR	10	160
SA + BR + AL + PLC	10	160
SA + BR + SC	10	155
AL + SC + BMR	10	150
BR + AL + SC + PLC	10	150
BR + BMR	9	145
SA + BR + AL	9	145
SA + PLC + BMR	9	145

**Table 3:** Coverage matrix *A*: $a_{ij}=1$ if route $R_{j}$ covers zone $Z_{i}$.

	$R_{1}$ (4 h)	$R_{2}$ (4 h)	$R_{3}$ (5 h)	$R_{4}$ (3 h)	$R_{5}$ (3 h)	$R_{6}$ (2 h)
** $Z_{1}$ **	1	0	1	0	1	0
** $Z_{2}$ **	1	1	0	0	0	0
** $Z_{3}$ **	0	1	1	0	0	0
** $Z_{4}$ **	0	1	0	1	1	0
** $Z_{5}$ **	0	0	1	1	0	1

**Table 4:** S-shaped and V-shaped transfer functions.

S-Shaped		V-Shaped	
Name	Equation	Name	Equation
S1	$T\left(d_{j}^{i}\right)=\frac{1}{1+e^{-2d_{j}^{i}}}$	V1	$T\left(d_{j}^{i}\right)=\left|\mathrm{erf}\left(\sqrt{\frac{\pi }{2}d_{j}^{i}}\right)\right|$
S2	$T\left(d_{j}^{i}\right)=\frac{1}{1+e^{-d_{j}^{i}}}$	V2	$T\left(d_{j}^{i}\right)=\left|\tanh(d_{j}^{i})\right|$
S3	$T\left(d_{j}^{i}\right)=\frac{1}{1+e^{-d_{j}^{i}/2}}$	V3	$T\left(d_{j}^{i}\right)=\left|\frac{d_{j}^{i}}{\sqrt{1+{\left(d_{j}^{i}\right)}^{2}}}\right|$
S4	$T\left(d_{j}^{i}\right)=\frac{1}{1+e^{-d_{j}^{i}/3}}$	V4	$T\left(d_{j}^{i}\right)=\left|\frac{2}{\pi }\arctan\left(\frac{\pi }{2}d_{j}^{i}\right)\right|$

**Table 5:** Instances used for parameter configuration (subset for KP, SCP, and USCP).

Instance	Type	Items (KP)	*M*	*N*	Density (%)	Optimum
knapPI_1_500_1000_1	KP	500	–	–	–	28,857
knapPI_1_1000_1000_1	KP	1000	–	–	–	54,503
knapPI_1_5000_1000_1	KP	5000	–	–	–	276,457
b1	SCP	–	300	3000	5.00	69
c1	SCP	–	400	4000	2.00	227
nre1	SCP	–	500	5000	10.00	29
u41	USCP	–	200	1000	2.00	38
uc1	USCP	–	400	4000	2.00	43
unrf1	USCP	–	500	5000	20.00	10

**Table 6:** Parameter selection at 100 iterations (population size = ten; averages over 31 runs).

Pop	Iter	Instance	Best	Worst	Average	Time (s)	Time (min)
KP (S1–STD, maximization)							
10	100	knapPI_1_500_1000_1	28,857	28,834	28,848.097	2.714	0.05
10	100	knapPI_1_1000_1000_1	54,451	54,064	54,303.710	5.863	0.10
10	100	knapPI_1_5000_1000_1	267,200	263,802	265,321.581	52.376	0.87
SCP (V3–ELIT, minimization)							
10	100	b1	69	71	70.000	63.140	1.05
10	100	c1	232	236	233.516	215.896	3.60
10	100	nre1	29	29	29.000	56.867	0.95
USCP (V3–ELIT, minimization)							
10	100	u41	38	41	39.774	10.229	0.17
10	100	uc1	44	46	44.645	71.331	1.19
10	100	unrf1	10	11	10.452	135.314	2.26

**Table 7:** S vs. V with binarization at 100 iterations (averages over 31 runs).

Problem	Instance	Transfer	Rule	Optimum	Avg. Value	Gap (%)/Time (s)
KP (maximization; Gap% = (Opt− Avg)/Opt ×100)						
KP	knapPI_1_500_1000_1	S1	STD	28,857	28,848.097	0.03%/2.714
KP	knapPI_1_500_1000_1	V1	STD	28,857	27,976.613	3.05%/0.727
KP	knapPI_1_1000_1000_1	S1	STD	54,503	54,303.710	0.37%/5.863
KP	knapPI_1_1000_1000_1	V1	STD	54,503	50,257.613	7.80%/1.328
KP	knapPI_1_5000_1000_1	S1	STD	276,457	265,321.581	4.03%/52.376
KP	knapPI_1_5000_1000_1	V1	STD	276,457	237,092.677	14.23%/7.010
SCP (minimization; Gap% = (Avg−Opt)/Opt ×100)						
SCP	b1	S3	ELIT	69	70.065	1.54%/73.290
SCP	b1	V3	ELIT	69	70.000	1.45%/63.140
SCP	c1	S3	ELIT	227	234.677	3.38%/249.704
SCP	c1	V3	ELIT	227	233.516	2.87%/215.896
SCP	nre1	S3	ELIT	29	29.065	0.22%/204.980
SCP	nre1	V3	ELIT	29	29.000	0.00%/56.867
USCP (minimization; Gap% = (Avg−Opt)/Opt ×100)						
USCP	u41	S3	ELIT	38	41.194	8.41%/14.809
USCP	u41	V3	ELIT	38	39.774	4.67%/10.229
USCP	uc1	S3	ELIT	43	47.484	10.42%/143.564
USCP	uc1	V3	ELIT	43	44.645	3.83%/71.331
USCP	unrf1	S3	ELIT	10	10.806	8.06%/184.001
USCP	unrf1	V3	ELIT	10	10.452	4.52%/135.314

**Table 8:** Configuration of parameters.

Parameter	Value	
All MH	Population size	10
	Iterations	100
	Independent runs	31
KP	Transfer functions	S1 – V1
	Method of discretization	STD
SCP – USCP	Transfer functions	S3 – V3
	Method of discretization	ELIT
PSO	$w_{min}$	0.1
	$w_{max}$	0.9
	$c_{1}$	2
	$c_{2}$	2
SBOA	CF	potentially decreases at 0
AOA	$MOP_{min}$	0.2
	$MOP_{max}$	1.0
	α	5
	μ	0.499

**Table 9:** Instances used for KP.

Instance	Number of Items	Optimum
knapPI_1_100_1000_1	100	9147
knapPI_1_200_1000_1	200	11,238
knapPI_1_500_1000_1	500	28,857
knapPI_1_1000_1000_1	1000	54,503
knapPI_1_2000_1000_1	2000	110,625
knapPI_1_5000_1000_1	5000	276,457
knapPI_1_10000_1000_1	10,000	563,647
knapPI_2_100_1000_1	100	1514
knapPI_2_200_1000_1	200	1634
knapPI_2_500_1000_1	500	4566
knapPI_2_1000_1000_1	1000	9052
knapPI_2_2000_1000_1	2000	18,051
knapPI_2_5000_1000_1	5000	44,356
knapPI_2_10000_1000_1	10,000	90,204
knapPI_3_100_1000_1	100	2397
knapPI_3_200_1000_1	200	2697
knapPI_3_500_1000_1	500	7117
knapPI_3_1000_1000_1	1000	14,390
knapPI_3_2000_1000_1	2000	28,919
knapPI_3_5000_1000_1	5000	72,505
knapPI_3_10000_1000_1	10,000	146,919

**Table 10:** Instances used for SCP.

Instance	M	N	Density (%)	Optimum
41	200	1000	2.00	429
51	200	2000	2.00	253
61	200	1000	5.00	138
A1	300	3000	2.00	253
B1	300	3000	5.00	69
C1	400	4000	2.00	227
D1	400	4000	5.00	60
NRE1	500	5000	10.00	29
NRF1	500	5000	20.00	14
NRG1	1000	10,000	2.00	176
NRH1	1000	10,000	5.00	63

**Table 11:** Instances used for USCP.

Instance	M	N	Density (%)	Optimum
U41	200	1000	2.00	38
U51	200	2000	2.00	34
U61	200	1000	5.00	21
UA1	300	3000	2.00	39
UB1	300	3000	5.00	22
UC1	400	4000	2.00	43
UD1	400	4000	5.00	24
UNRE1	500	5000	10.00	17
UNRF1	500	5000	20.00	10
UNRG1	1000	10,000	2.00	61
UNRH1	1000	10,000	5.00	34
CLR10	511	210	12.30	25
CYC08	1792	1024	0.40	344

**Table 12:** Fitness results per KP instance using S1-STD binarization.

MH	Instance	Opt.	Best	Worst	Avg. Fitness	Std. Fitness	RPD
POA	knapPI_1_100_1000_1	9147	9147.000	9147.000	9147.000	0.000	0.000
PSO			9147.000	9147.000	9147.000	0.000	0.000
SBOA			9147.000	9147.000	9147.000	0.000	0.000
AOA			9147.000	9147.000	9147.000	0.000	0.000
POA	knapPI_1_200_1000_1	11,238	11,238.000	11,238.000	11,238.000	0.000	0.000
PSO			11,238.000	11,238.000	11,238.000	0.000	0.000
SBOA			11,238.000	11,238.000	11,238.000	0.000	0.000
AOA			11,238.000	11,238.000	11,238.000	0.000	0.000
POA	knapPI_1_500_1000_1	28,857	28,857.000	28,834.000	28,848.097	11.388	0.031
PSO			28,857.000	28,834.000	28,846.613	11.630	0.036
SBOA			28,857.000	28,834.000	28,849.548	10.998	0.026
AOA			28,834.000	28,360.000	28,599.452	133.473	0.893
POA	knapPI_1_1000_1000_1	54,503	54,451.000	54,064.000	54,303.710	83.397	0.366
PSO			54,481.000	53,945.000	54,225.581	119.134	0.509
SBOA			54,503.000	54,102.000	54,286.839	111.431	0.397
AOA			53,197.000	51,786.000	52,317.581	371.439	4.010
POA	knapPI_1_2000_1000_1	110,625	109,619.000	108,005.000	108,681.355	391.045	1.757
PSO			109,676.000	107,805.000	108,486.968	517.345	1.933
SBOA			109,474.000	108,032.000	108,611.903	308.203	1.820
AOA			104,085.000	100,624.000	102,140.452	928.618	7.670
POA	knapPI_1_5000_1000_1	276,457	267,200.000	263,802.000	265,321.581	871.781	4.028
PSO			266,224.000	263,666.000	264,813.480	712.781	4.211
SBOA			268,586.000	264,142.000	265,636.000	1011.621	3.914
AOA			247,200.000	242,275.000	244,586.258	1192.176	11.528
POA	knapPI_1_10000_1000_1	563,647	535,652.000	528,828.000	531,830.742	1805.202	5.644
PSO			534,108.000	528,154.000	530,036.774	1290.393	5.962
SBOA			536,441.000	529,071.000	531,588.581	1781.996	5.688
AOA			485,986.000	477,145.000	481,075.839	2125.044	14.651
POA	knapPI_2_100_1000_1	1514	1512.000	1512.000	1512.000	0.000	0.000
PSO			1512.000	1512.000	1512.000	0.000	0.000
SBOA			1512.000	1512.000	1512.000	0.000	0.132
AOA			1512.000	1512.000	1512.000	0.000	0.132
POA	knapPI_2_200_1000_1	1634	1634.000	1634.000	1634.000	0.000	0.000
PSO			1634.000	1634.000	1634.000	0.000	0.000
SBOA			1634.000	1634.000	1634.000	0.000	0.000
AOA			1634.000	1634.000	1634.000	0.000	0.000
POA	knapPI_2_500_1000_1	4566	4566.000	4553.000	4560.323	4.805	0.031
PSO			4566.000	4552.000	4558.226	4.617	0.036
SBOA			4566.000	4554.000	4559.871	4.425	0.134
AOA			4566.000	4547.000	4552.742	3.421	0.290
POA	knapPI_2_1000_1000_1	9052	9051.000	9026.000	9046.194	4.086	0.067
PSO			9051.000	9033.000	9044.516	4.774	0.084
SBOA			9051.000	9039.000	9047.032	3.297	0.055
AOA			9015.000	8913.000	8963.516	23.475	0.978
POA	knapPI_2_2000_1000_1	18,051	18,012.000	17,911.000	17,957.258	24.223	0.519
PSO			17,971.000	17,900.000	17,932.387	21.750	0.657
SBOA			18,026.000	17,886.000	17,958.065	34.712	0.515
AOA			17,681.000	17,472.000	17,567.065	52.484	2.681
POA	knapPI_2_5000_1000_1	44,356	43,889.000	43,592.000	43,757.800	61.292	1.348
PSO			43,872.000	43,596.000	226.200	74.717	1.446
SBOA			43,868.000	43,612.000	43,745.452	44.969	1.376
AOA			42,630.000	42,265.000	42,421.871	84.446	4.360
POA	knapPI_2_10000_1000_1	90,204	88,613.000	88,002.000	88275.290	149.288	2.138
PSO			88,403.000	87,854.000	88,134.387	135.942	2.294
SBOA			88,484.000	88,112.000	88,263.161	108.102	2.152
AOA			85,108.000	84,646.000	84,850.323	140.986	5.935

**Table 13:** Fitness results per KP instance using S1-STD binarization.

MH	Instance	Opt.	Best	Worst	Avg. Fitness	Std. Fitness	RPD
POA	knapPI_3_100_1000_1	2397	2397.000	2397.000	2397.000	0.000	0.000
PSO			2397.000	2396.000	2396.935	0.250	0.002
SBOA			2397.000	2397.000	2397.000	0.000	0.000
AOA			2397.000	2396.000	2396.968	0.177	0.001
POA	knapPI_3_200_1000_1	2697	2697.000	2697.000	2697.000	0.000	0.000
PSO			2697.000	2697.000	2697.000	0.000	0.000
SBOA			2697.000	2697.000	2697.000	0.000	0.000
AOA			2697.000	2697.000	2697.000	0.000	0.000
POA	knapPI_3_500_1000_1	7117	7117.000	7116.000	7116.968	0.180	0.000
PSO			7117.000	7117.000	7117.000	0.000	0.000
SBOA			7117.000	7117.000	7117.000	0.000	0.000
AOA			7115.000	7012.000	7021.871	23.989	1.337
POA	knapPI_3_1000_1000_1	14,390	14,388.000	14,287.000	14,298.613	28.659	0.632
PSO			14,387.000	14,275.000	14,294.226	24.709	0.664
SBOA			14,390.000	14,287.000	14,305.484	36.384	0.587
AOA			14,087.000	13,877.000	13,938.968	65.543	3.134
POA	knapPI_3_2000_1000_1	28,919	28,704.000	28,418.000	28,555.290	63.345	1.258
PSO			28,718.000	28,318.000	28,473.710	84.328	1.540
SBOA			28,814.000	28,417.000	28,534.387	76.643	1.330
AOA			27,606.000	27,109.000	27,245.871	113.777	5.786
POA	knapPI_3_5000_1000_1	72,505	70,593.000	70,099.000	70,360.258	126.873	2.959
PSO			70,603.000	69,905.000	70,231.419	159.188	3.135
SBOA			70,694.000	70,199.000	70,377.129	133.900	2.935
AOA			67,701.000	65,996.000	66,383.323	322.218	8.443
POA	knapPI_3_10000_1000_1	146,919	141,818.000	140,618.000	141,075.548	266.529	3.977
PSO			141,215.000	140,507.000	140,827.323	211.712	4.146
SBOA			141,317.000	140,716.000	141,052.226	165.625	3.993
AOA			132,716.000	131,313.000	132,050.871	320.990	10.120

**Table 14:** Times per KP instance (S1-STD).

MH	Instance	Min. Time (s)	Max. Time (s)	Avg. Time (s)	Std. Time (s)
POA	knapPI_1_100_1000_1	0.531	0.750	0.620	0.059
PSO		0.141	0.281	0.204	0.035
SBOA		0.453	0.672	0.515	0.051
AOA		0.437	0.609	0.509	0.040
POA	knapPI_1_200_1000_1	0.984	1.375	1.142	0.103
PSO		0.328	0.500	0.404	0.045
SBOA		0.859	1.218	1.028	0.088
AOA		0.828	1.156	0.975	0.088
POA	knapPI_1_500_1000_1	2.203	3.530	2.714	0.389
PSO		0.765	1.172	0.908	0.108
SBOA		2.249	3.046	2.536	0.215
AOA		2.125	2.796	2.361	0.177
POA	knapPI_1_1000_1000_1	4.905	6.623	5.863	0.457
PSO		1.531	2.328	1.877	0.199
SBOA		5.061	6.608	5.642	0.380
AOA		4.327	5.264	4.796	0.229
POA	knapPI_1_2000_1000_1	11.482	16.059	13.855	1.352
PSO		3.202	4.561	3.814	0.371
SBOA		12.247	13.950	12.969	0.496
AOA		9.716	10.919	10.158	0.334
POA	knapPI_1_5000_1000_1	43.459	66.500	52.376	5.814
PSO		8.873	13.153	10.841	1.150
SBOA		48.989	52.753	50.840	1.039
AOA		29.821	32.945	31.214	0.816
POA	knapPI_1_10000_1000_1	137.686	197.750	166.984	13.539
PSO		22.197	35.952	27.593	3.218
SBOA		152.371	163.685	160.279	2.062
AOA		78.404	85.964	80.741	1.391
POA	knapPI_2_100_1000_1	0.484	0.734	0.587	0.067
PSO		0.141	0.219	0.175	0.024
SBOA		0.484	0.687	0.563	0.056
AOA		0.453	0.625	0.514	0.053
POA	knapPI_2_200_1000_1	0.906	1.500	1.154	0.147
PSO		0.312	0.469	0.381	0.047
SBOA		0.906	1.265	1.048	0.088
AOA		0.828	1.203	0.983	0.101
POA	knapPI_2_500_1000_1	2.265	3.640	2.762	0.392
PSO		0.734	1.156	0.885	0.113
SBOA		2.234	3.077	2.554	0.218
AOA		2.125	2.937	2.385	0.187
POA	knapPI_2_1000_1000_1	4.983	7.358	5.931	0.764
PSO		1.500	2.218	1.838	0.208
SBOA		5.093	6.592	5.723	0.352
AOA		4.530	5.358	4.841	0.226
POA	knapPI_2_2000_1000_1	11.544	17.418	14.248	1.618
PSO		3.124	5.077	3.780	0.459
SBOA		12.294	14.481	13.397	0.532
AOA		9.279	11.044	10.305	0.419
POA	knapPI_2_5000_1000_1	44.927	68.406	54.793	5.535
PSO		8.920	13.466	10.872	1.199
SBOA		51.113	56.206	52.754	1.074
AOA		30.477	34.351	32.014	0.913
POA	knapPI_2_10000_1000_1	141.201	213.872	173.623	17.640
PSO		22.573	37.429	27.534	3.419
SBOA		159.369	169.898	163.786	2.620
AOA		80.200	88.261	83.010	1.671

**Table 15:** Times per KP instance (S1-STD).

MH	Instance	Min. Time (s)	Max. Time (s)	Avg. Time (s)	Std. Time (s)
POA	knapPI_3_100_1000_1	0.469	0.734	0.598	0.072
PSO		0.141	0.203	0.168	0.017
SBOA		0.453	0.672	0.543	0.053
AOA		0.422	0.625	0.511	0.049
POA	knapPI_3_200_1000_1	0.922	1.453	1.111	0.123
PSO		0.297	0.453	0.373	0.040
SBOA		0.875	1.281	1.019	0.097
AOA		0.844	1.250	0.964	0.084
POA	knapPI_3_500_1000_1	2.296	2.937	2.565	0.164
PSO		0.719	0.953	0.806	0.064
SBOA		2.281	2.890	2.516	0.161
AOA		1.921	2.656	2.304	0.202
POA	knapPI_3_1000_1000_1	4.796	7.483	5.795	0.737
PSO		1.437	2.421	1.774	0.291
SBOA		5.046	6.374	5.658	0.316
AOA		4.468	5.202	4.778	0.181
POA	knapPI_3_2000_1000_1	11.247	17.152	13.547	1.544
PSO		3.093	5.171	3.747	0.498
SBOA		12.060	13.716	12.806	0.488
AOA		9.467	10.607	10.113	0.311
POA	knapPI_3_5000_1000_1	47.536	51.816	49.705	0.991
PSO		8.670	13.637	10.764	1.205
SBOA		48.286	53.972	50.299	1.232
AOA		29.446	33.320	31.124	0.798
POA	knapPI_3_10000_1000_1	135.046	198.672	163.974	16.361
PSO		21.979	32.774	26.913	3.364
SBOA		152.683	164.227	157.780	2.722
AOA		76.888	83.949	79.855	1.498

**Table 16:** Fitness results per KP instance using V1-STD binarization.

MH	Instance	Opt.	Best	Worst	Avg. Fitness	Std. Fitness	RPD
POA	knapPI_1_100_1000_1	9147	9147.000	8817.000	9012.935	140.421	1.466
PSO			9147.000	8382.000	8806.355	211.194	3.723
SBOA			9147.000	8817.000	9004.161	159.063	1.562
AOA			9147.000	8259.000	8769.613	219.035	4.126
POA	knapPI_1_200_1000_1	11,238	11,238.000	11,227.000	11,227.710	2.747	0.094
PSO			11,238.000	9996.000	10,781.935	303.375	4.058
SBOA			11,238.000	11,227.000	11,227.355	1.944	0.095
AOA			11,223.000	10,049.000	10,689.710	327.592	4.879
POA	knapPI_1_500_1000_1	28,857	28,834.000	27,191.000	27,976.613	384.620	3.051
PSO			26,826.000	24,015.000	25,755.484	642.632	10.747
SBOA			28,834.000	27,563.000	28,005.903	342.911	2.949
AOA			27,434.000	23,935.000	25,686.355	770.764	10.987
POA	knapPI_1_1000_1000_1	54,503	52,068.000	49,104.000	50,257.613	745.510	7.790
PSO			48,194.000	43,592.000	45,806.516	1265.735	15.955
SBOA			52,032.000	49,203.000	50,175.387	708.908	7.940
AOA			47,866.000	43,653.000	45,646.774	1097.034	16.249
POA	knapPI_1_2000_1000_1	110,625	100,075.000	96,693.000	98,328.258	920.659	11.116
PSO			92,932.000	85,064.000	89,699.968	1907.719	18.916
SBOA			100,160.000	96,088.000	98,177.677	1053.376	11.252
AOA			92,339.000	87,116.000	89,570.129	1436.595	19.033
POA	knapPI_1_5000_1000_1	276,457	244,096.000	232,686.000	237,092.677	2242.510	14.239
PSO			220,459.000	209,969.000	214,835.774	2635.139	22.290
SBOA			240,936.000	234,072.000	236,346.774	1577.169	14.509
AOA			218,553.000	210,698.000	215,019.258	2209.594	22.223
POA	knapPI_1_10000_1000_1	563,647	478,675.000	467,908.000	470,893.194	2429.439	16.455
PSO			432,686.000	417,866.000	424,146.677	3800.358	24.749
SBOA			480,921.000	464,959.000	471,139.581	3564.162	16.412
AOA			435,222.000	415,563.000	425,328.387	4867.362	24.540
POA	knapPI_2_100_1000_1	1514	1512.000	1496.000	1505.452	6.647	0.564
PSO			1512.000	1449.000	1493.677	13.850	1.343
SBOA			1512.000	1481.000	1504.613	7.210	0.620
AOA			1512.000	1430.000	1489.516	16.008	1.617
POA	knapPI_2_200_1000_1	1634	1634.000	1610.000	1624.484	6.855	0.583
PSO			1634.000	1585.000	1610.419	14.056	1.443
SBOA			1634.000	1604.000	1621.710	7.751	0.752
AOA			1633.000	1560.000	1608.097	17.713	1.585
POA	knapPI_2_500_1000_1	4566	4551.000	4465.000	4534.194	26.513	0.698
PSO			4497.000	4286.000		61.927	3.815
SBOA			4566.000	4400.000	4520.871	44.660	0.988
AOA			4484.000	4241.000		62.950	4.232
POA	knapPI_2_1000_1000_1	9052	8934.000	8448.000	8649.806	108.219	4.443
PSO			8708.000	8382.000	8518.484	85.428	5.893
SBOA			8808.000	8459.000	8627.613	84.077	4.688
AOA			8672.000	8375.000	8527.419	64.538	5.795
POA	knapPI_2_2000_1000_1	18,051	16,877.000	16,428.000	16,627.710	111.241	7.884
PSO			17,184.000	16,323.000	16,674.355	216.325	7.626
SBOA			17,013.000	16,351.000	16,666.226	140.864	7.671
AOA			17,015.000	16,325.000	16,664.903	176.993	7.679
POA	knapPI_2_5000_1000_1	44,356	40,583.000	39,900.000	40,295.129	188.227	9.155
PSO			40,900.000	39,781.000	40,331.806	259.406	9.072
SBOA			40,893.000	40,015.000	40,372.290	209.545	8.981
AOA			40,916.000	39,963.000	40,374.129	235.313	8.977
POA	knapPI_2_10000_1000_1	90,204	81,559.000	80,194.000	80,812.774	325.683	10.412
PSO			81,916.000	80,070.000	80,954.355	438.732	10.254
SBOA			81,785.000	80,073.000	80,734.677	374.709	10.498
AOA			81,873.000	79,881.000	80,857.387	476.805	10.362

**Table 17:** Fitness results per KP instance using V1-STD binarization.

MH	Instance	Opt.	Best	Worst	Avg. Fitness	Std. Fitness	RPD
POA	knapPI_3_100_1000_1	2397	2397.000	2375.000	2391.226	6.566	0.240
PSO			2390.000	2265.000	2302.903	37.669	3.925
SBOA			2397.000	2375.000	2388.484	7.152	0.355
AOA			2396.000	2237.000	2297.161	36.929	4.165
POA	knapPI_3_200_1000_1	2697	2697.000	2672.000	2688.903	7.445	0.301
PSO			2696.000	2487.000	2633.194	55.404	2.366
SBOA			2697.000	2649.000	2679.161	12.347	0.661
AOA			2697.000	2573.000	2629.129	48.122	2.517
POA	knapPI_3_500_1000_1	7117	6989.000	6661.000	6823.742	83.349	4.122
PSO			6913.000	6495.000	6649.935	109.175	6.563
SBOA			6938.000	6614.000	6758.032	77.142	5.044
AOA			6808.000	6410.000	6593.484	101.348	7.356
POA	knapPI_3_1000_1000_1	14,390	13,564.000	12,881.000	13,187.323	162.640	8.358
PSO			13,190.000	12,586.000	12,899.129	183.281	10.361
SBOA			13,589.000	12,879.000	13,142.839	190.814	8.667
AOA			13,385.000	12,582.000	12,946.484	192.586	10.031
POA	knapPI_3_2000_1000_1	28,919	26,072.000	25,132.000	25,537.097	240.077	11.695
PSO			25,618.000	24,618.000	25,107.355	238.308	13.180
SBOA			26,468.000	25,015.000	25,433.290	292.369	12.053
AOA			25,510.000	24,417.000	25,078.129	271.899	13.281
POA	knapPI_3_5000_1000_1	72,505	62,807.000	61,104.000	61,690.129	405.209	14.916
PSO			62,603.000	61,000.000	61,484.516	436.343	15.200
SBOA			62,700.000	60,697.000	61,683.290	396.871	14.925
AOA			63,601.000	60,701.000	61,677.581	529.037	14.933
POA	knapPI_3_10000_1000_1	146,919	124,419.000	121,715.000	122,723.645	541.988	16.469
PSO			124,216.000	121,516.000	122,845.258	677.189	16.386
SBOA			125,019.000	121,716.000	122,749.742	726.676	16.451
AOA			123,519.000	121,218.000	122,412.968	481.210	16.680

**Table 18:** Times per KP instance (V1-STD).

MH	Instance	Min. Time (s)	Max. Time (s)	Avg. Time (s)	Std. Time (s)
POA	knapPI_1_100_1000_1	0.214	0.297	0.248	0.017
PSO		0.071	0.104	0.078	0.009
SBOA		0.172	0.266	0.222	0.027
AOA		0.312	0.516	0.390	0.047
POA	knapPI_1_200_1000_1	0.332	0.445	0.370	0.028
PSO		0.127	0.182	0.150	0.018
SBOA		0.250	0.422	0.315	0.045
AOA		0.594	0.859	0.719	0.076
POA	knapPI_1_500_1000_1	0.634	0.828	0.727	0.045
PSO		0.313	0.435	0.369	0.031
SBOA		0.500	0.812	0.667	0.071
AOA		1.453	1.937	1.717	0.124
POA	knapPI_1_1000_1000_1	1.188	1.445	1.328	0.069
PSO		0.642	0.856	0.715	0.051
SBOA		1.000	1.422	1.173	0.099
AOA		2.937	3.718	3.253	0.199
POA	knapPI_1_2000_1000_1	2.460	2.811	2.589	0.087
PSO		1.264	1.630	1.410	0.093
SBOA		2.046	2.734	2.334	0.150
AOA		5.842	7.201	6.342	0.366
POA	knapPI_1_5000_1000_1	6.601	7.548	7.010	0.220
PSO		3.237	4.017	3.481	0.192
SBOA		5.967	6.920	6.360	0.261
AOA		14.840	17.246	15.796	0.533
POA	knapPI_1_10000_1000_1	15.691	17.662	16.444	0.447
PSO		6.632	7.812	7.080	0.266
SBOA		14.125	15.672	14.838	0.351
AOA		30.462	33.367	31.935	0.892
POA	knapPI_2_100_1000_1	0.208	0.269	0.234	0.015
PSO		0.071	0.100	0.079	0.009
SBOA		0.156	0.281	0.211	0.032
AOA		0.344	0.500	0.402	0.039
POA	knapPI_2_200_1000_1	0.307	0.407	0.345	0.026
PSO		0.124	0.182	0.145	0.017
SBOA		0.234	0.375	0.310	0.034
AOA		0.594	1.015	0.731	0.081
POA	knapPI_2_500_1000_1	0.649	0.785	0.719	0.033
PSO		0.351	0.502	0.408	0.038
SBOA		0.531	0.812	0.637	0.065
AOA		1.531	2.031	1.676	0.126
POA	knapPI_2_1000_1000_1	1.205	1.461	1.312	0.077
PSO		0.623	0.817	0.711	0.051
SBOA		0.953	1.359	1.147	0.100
AOA		2.906	3.749	3.255	0.210
POA	knapPI_2_2000_1000_1	2.308	2.798	2.506	0.107
PSO		1.269	1.748	1.398	0.078
SBOA		1.890	2.562	2.142	0.148
AOA		5.842	6.967	6.359	0.295
POA	knapPI_2_5000_1000_1	6.214	7.091	6.549	0.187
PSO		3.217	4.005	3.483	0.179
SBOA		5.311	6.217	5.659	0.249
AOA		14.450	17.105	15.705	0.660
POA	knapPI_2_10000_1000_1	14.892	16.552	15.568	0.420
PSO		6.659	7.703	7.121	0.225
SBOA		12.294	13.684	13.063	0.362
AOA		29.993	33.601	31.717	0.858

**Table 19:** Times per KP instance (V1-STD).

MH	Instance	Min. Time (s)	Max. Time (s)	Avg. Time (s)	Std. Time (s)
POA	knapPI_3_100_1000_1	0.219	0.307	0.256	0.021
PSO		0.071	0.097	0.076	0.007
SBOA		0.172	0.281	0.223	0.025
AOA		0.344	0.500	0.389	0.036
POA	knapPI_3_200_1000_1	0.319	0.398	0.358	0.021
PSO		0.125	0.178	0.143	0.014
SBOA		0.250	0.406	0.326	0.035
AOA		0.594	0.937	0.734	0.091
POA	knapPI_3_500_1000_1	0.654	0.887	0.739	0.052
PSO		0.330	0.447	0.374	0.031
SBOA		0.547	0.828	0.653	0.068
AOA		1.531	1.968	1.694	0.099
POA	knapPI_3_1000_1000_1	1.173	1.462	1.346	0.069
PSO		0.640	0.824	0.711	0.044
SBOA		1.062	1.640	1.235	0.124
AOA		2.906	3.655	3.253	0.194
POA	knapPI_3_2000_1000_1	2.420	2.779	2.606	0.099
PSO		1.233	1.531	1.392	0.069
SBOA		2.125	2.827	2.388	0.152
AOA		5.827	7.014	6.332	0.290
POA	knapPI_3_5000_1000_1	6.655	7.571	7.067	0.263
PSO		3.153	3.787	3.487	0.171
SBOA		6.014	7.123	6.525	0.297
AOA		14.668	17.246	15.891	0.527
POA	knapPI_3_10000_1000_1	16.410	17.772	16.894	0.322
PSO		6.730	7.653	7.090	0.248
SBOA		14.887	16.543	15.650	0.490
AOA		30.024	34.101	31.540	0.878

**Table 20:** Fitness results per SCP instance using V3-ELIT binarization.

MH	Instance	Opt.	Best	Worst	Avg. Fitness	Std. Fitness	RPD
POA	41	429	433.000	433.000	433.000	0.000	0.930
PSO			433.000	433.000	433.000	0.000	0.930
SBOA			433.000	434.000	433.194	0.395	0.978
AOA			435.000	439.000	437.194	0.858	1.910
POA	51	253	267.000	269.000	267.419	0.564	5.697
PSO			267.000	269.000	267.419	0.765	5.696
SBOA			263.000	269.000	267.097	0.893	5.572
AOA			261.000	271.000	269.161	1.687	6.388
POA	61	138	141.000	145.000	141.935	1.632	2.849
PSO			141.000	145.000	142.032	1.663	2.919
SBOA			141.000	144.000	141.290	0.632	2.384
AOA			141.000	147.000	142.839	1.628	3.506
POA	a1	253	257.000	257.000	257.000	0.000	1.580
PSO			257.000	258.000	257.097	0.301	1.619
SBOA			257.000	259.000	257.516	0.615	1.785
AOA			259.000	266.000	262.419	1.737	3.723
POA	b1	69	69.000	71.000	70.000	0.966	1.450
PSO			69.000	72.000	70.452	1.028	2.105
SBOA			69.000	71.000	70.000	0.622	1.449
AOA			69.000	72.000	70.290	0.811	1.870
POA	c1	227	232.000	236.000	233.516	0.769	2.867
PSO			231.000	236.000	233.871	0.922	3.023
SBOA			234.000	236.000	234.774	0.551	3.425
AOA			237.000	242.000	239.419	1.212	5.471
POA	d1	60	60.000	63.000	61.871	0.885	3.118
PSO			61.000	65.000	62.032	1.048	3.388
SBOA			61.000	62.000	61.645	0.478	2.742
AOA			61.000	64.000	62.258	0.879	3.763
POA	nre1	29	29.000	29.000	29.000	0.000	0.000
PSO			29.000	29.000	29.000	0.000	0.000
SBOA			29.000	29.000	29.000	0.000	0.000
AOA			29.000	29.000	29.000	0.000	0.000
POA	nrf1	14	14.000	15.000	14.032	0.180	0.230
PSO			14.000	15.000	14.161	0.374	1.152
SBOA			14.000	14.000	14.000	0.000	0.000
AOA			14.000	15.000	14.032	0.177	0.230
POA	nrg1	176	179.000	186.000	181.968	1.602	3.391
PSO			179.000	185.000	181.839	1.551	3.318
SBOA			180.000	184.000	181.742	1.015	3.262
AOA			183.000	189.000	186.645	1.514	6.048
POA	nrh1	63	64.000	68.000	65.613	1.022	4.146
PSO			64.000	68.000	65.613	1.174	4.147
SBOA			64.000	67.000	64.903	0.893	3.021
AOA			65.000	69.000	66.742	0.879	5.940

**Table 21:** Times per SCP instance (V3-ELIT).

MH	Instance	Min. Time (s)	Max. Time (s)	Avg. Time (s)	Std. Time (s)
POA	41	18.886	20.995	20.050	0.533
PSO		15.075	15.403	15.280	0.087
SBOA		16.481	20.558	18.276	0.942
AOA		13.325	16.610	14.588	0.750
POA	51	31.446	35.632	33.233	1.075
PSO		21.870	22.745	22.238	0.199
SBOA		28.134	43.521	32.714	2.750
AOA		21.448	27.806	24.129	1.757
POA	61	12.747	15.856	14.362	0.819
PSO		9.779	10.826	10.260	0.247
SBOA		12.075	16.605	13.845	1.103
AOA		9.092	13.497	10.403	1.001
POA	a1	85.902	98.742	93.412	2.839
PSO		47.379	48.676	47.880	0.275
SBOA		84.886	109.178	95.718	5.624
AOA		54.518	66.375	59.737	3.258
POA	b1	57.065	67.625	63.140	2.380
PSO		29.712	32.086	30.666	0.588
r		59.377	73.717	66.340	3.130
AOA		35.211	45.692	39.095	2.461
POA	c1	200.484	228.774	215.896	6.847
PSO		89.744	93.806	91.744	0.875
SBOA		199.719	235.429	216.854	8.214
AOA		121.597	147.590	136.698	6.073
POA	d1	128.407	144.872	137.608	4.022
PSO		60.772	64.023	62.214	0.727
SBOA		131.375	155.385	140.616	5.884
AOA		73.420	95.571	82.526	4.730
POA	nre1	54.331	59.564	56.867	1.462
PSO		8.779	9.123	8.964	0.083
SBOA		179.161	214.090	194.242	7.053
AOA		94.572	111.552	104.009	4.155
POA	nrf1	81.731	90.104	84.937	2.060
PSO		8.514	9.357	8.969	0.195
SBOA		179.146	215.949	203.102	9.491
AOA		82.871	110.724	90.278	5.577
POA	nrg1	124.814	136.874	132.288	3.018
PSO		110.989	113.958	112.398	0.822
SBOA		1689.564	1802.659	1743.972	27.850
AOA		1046.536	1123.401	1085.385	19.897
POA	nrh1	141.217	143.060	142.019	0.437
PSO		75.701	76.295	75.986	0.134
SBOA		1085.975	1200.794	1147.707	29.547
AOA		609.077	666.186	635.279	13.771

**Table 22:** Fitness results per SCP instance using S3-ELIT binarization.

MH	Instance	Opt.	Best	Worst	Avg. Fitness	Std. Fitness	RPD
POA	41	429	433.000	438.000	434.323	1.759	1.241
PSO			433.000	437.000	434.000	1.317	1.165
SBOA			434.000	437.000	435.032	0.782	1.406
AOA			433.000	438.000	434.710	1.300	1.331
POA	51	253	256.000	269.000	267.323	2.227	5.659
PSO			257.000	275.000	267.710	2.559	5.813
SBOA			267.000	270.000	268.677	0.736	6.197
AOA			267.000	269.000	267.645	0.650	5.789
POA	61	138	141.000	145.000	143.258	1.879	3.808
PSO			141.000	146.000	143.903	1.680	4.276
SBOA			141.000	143.000	141.871	0.491	2.805
AOA			140.000	146.000	142.968	1.926	3.600
POA	a1	253	257.000	263.000	258.097	1.300	2.015
PSO			257.000	263.000	258.355	1.684	2.117
SBOA			257.000	262.000	259.774	1.038	2.678
AOA			257.000	262.000	258.645	1.151	2.231
POA	b1	69	69.000	72.000	70.065	1.124	1.544
PSO			69.000	72.000	70.452	1.060	2.105
SBOA			69.000	71.000	70.355	0.698	1.964
AOA			69.000	72.000	70.290	1.053	1.870
POA	c1	227	232.000	237.000	234.677	1.107	3.378
PSO			232.000	237.000	234.581	1.259	3.336
SBOA			235.000	239.000	236.839	0.954	4.334
AOA			234.000	238.000	235.613	1.127	3.794
POA	d1	60	60.000	64.000	61.839	0.898	3.064
PSO			60.000	65.000	62.000	1.125	3.333
SBOA			61.000	63.000	62.032	0.538	3.387
AOA			60.000	66.000	62.129	1.100	3.548
POA	nre1	29	29.000	30.000	29.065	0.250	0.223
PSO			29.000	30.000	29.032	0.180	0.111
SBOA			29.000	29.000	29.000	0.000	0.000
AOA			29.000	30.000	29.161	0.368	0.556
POA	nrf1	14	14.000	15.000	14.194	0.402	1.382
PSO			14.000	15.000	14.161	0.374	1.152
SBOA			14.000	14.000	14.000	0.000	0.000
AOA			14.000	15.000	14.161	0.368	1.152
POA	nrg1	176	180.000	186.000	182.452	1.710	3.666
PSO			180.000	186.000	182.871	1.607	3.905
SBOA			181.000	186.000	184.290	1.083	4.710
AOA			181.000	189.000	183.548	1.757	4.289
POA	nrh1	63	64.000	69.000	66.290	1.216	5.222
PSO			64.000	68.000	66.387	0.989	5.376
SBOA			64.000	68.000	66.226	0.906	5.120
AOA			64.000	69.000	66.355	1.094	5.325

**Table 23:** Times per SCP instance (S3-ELIT).

MH	Instance	Min. Time (s)	Max. Time (s)	Avg. Time (s)	Std. Time (s)
POA	41	19.093	26.774	22.355	2.202
PSO		18.370	19.980	19.111	0.427
SBOA		19.460	23.214	20.966	1.059
AOA		10.373	13.278	11.554	0.699
POA	51	32.485	44.239	37.369	2.973
PSO		27.891	29.238	28.484	0.354
SBOA		31.946	43.802	36.424	2.616
AOA		16.481	21.354	18.800	1.473
POA	61	13.540	18.838	15.811	1.418
PSO		11.847	12.771	12.409	0.219
SBOA		13.356	19.308	14.960	1.253
AOA		7.529	10.435	8.601	0.721
POA	a1	96.136	131.866	111.999	8.634
PSO		60.413	62.840	61.599	0.616
SBOA		97.774	117.894	107.362	5.107
AOA		36.976	52.425	43.538	3.925
POA	b1	64.978	84.457	73.290	4.733
PSO		36.273	38.049	37.078	0.361
SBOA		61.814	79.919	72.885	3.885
AOA		26.119	34.242	30.365	2.080
POA	c1	223.648	282.106	249.704	12.555
PSO		114.098	117.440	115.643	0.896
SBOA		228.337	257.940	243.520	7.216
AOA		85.886	107.272	95.005	5.114
POA	d1	139.768	174.884	156.284	8.726
PSO		68.120	71.813	69.993	0.848
SBOA		140.202	170.382	152.485	6.623
AOA		54.487	66.469	60.205	3.187
POA	nre1	186.188	232.270	204.980	10.529
PSO		82.612	86.093	84.121	0.726
SBOA		194.736	219.964	210.201	6.276
AOA		72.046	93.384	80.246	5.155
POA	nrf1	184.183	212.649	199.108	7.736
PSO		71.910	75.820	74.020	0.918
SBOA		182.270	219.121	202.481	7.891
AOA		65.860	82.121	73.664	3.556
POA	nrg1	1810.810	2070.819	1959.459	63.847
PSO		5665.184	5890.245	5781.635	57.955
SBOA		1889.903	1994.013	1946.354	26.387
AOA		711.511	777.449	742.672	18.612
POA	nrh1	1114.931	1273.890	1211.601	39.025
PSO		3047.762	3234.057	3134.413	44.761
SBOA		1175.110	1265.325	1212.904	23.259
AOA		373.846	480.090	438.872	25.540

**Table 24:** Fitness results per USCP instance using V3-ELIT binarization.

MH	Instance	Opt.	Best	Worst	Avg. Fitness	Std. Fitness	RPD
POA	u41	38	38.000	41.000	39.774	0.762	4.666
PSO			39.000	41.000	39.742	0.682	4.581
SBOA			39.000	41.000	39.774	0.551	4.669
AOA			38.000	41.000	40.032	0.782	5.348
POA	u51	34	35.000	37.000	35.935	0.680	5.690
PSO			35.000	37.000	36.000	0.577	5.880
SBOA			35.000	36.000	35.581	0.493	4.649
AOA			35.000	37.000	35.935	0.435	5.693
POA	ua1	38	40.000	41.000	40.387	0.495	6.278
PSO			40.000	42.000	40.903	0.539	7.636
SBOA			40.000	41.000	40.097	0.296	5.518
AOA			39.000	42.000	40.806	0.737	7.385
POA	ub1	22	22.000	23.000	22.806	0.402	3.669
PSO			22.000	24.000	22.839	0.523	3.815
SBOA			22.000	23.000	22.290	0.454	1.320
AOA			22.000	24.000	23.065	0.353	4.839
POA	uc1	43	44.000	46.000	44.645	0.608	3.827
PSO			44.000	47.000	45.129	0.670	4.952
SBOA			43.000	45.000	44.258	0.566	2.926
AOA			44.000	46.000	45.484	0.561	5.776
POA	ud1	24	25.000	26.000	25.323	0.475	5.512
PSO			25.000	26.000	25.645	0.486	6.854
SBOA			25.000	26.000	25.032	0.177	4.301
AOA			25.000	26.000	25.645	0.478	6.855
POA	unre1	16	17.000	18.000	17.097	0.301	6.855
PSO			17.000	18.000	17.161	0.374	7.258
SBOA			17.000	17.000	17.000	0.000	6.250
AOA			17.000	18.000	17.129	0.335	7.056
POA	unrf1	10	10.000	11.000	10.452	0.506	4.516
PSO			10.000	11.000	10.355	0.486	3.548
SBOA			10.000	11.000	10.032	0.177	0.323
AOA			10.000	11.000	10.355	0.478	3.548
POA	unrg1	60	62.000	63.000	62.710	0.461	4.515
PSO			62.000	65.000	63.871	0.718	6.453
SBOA			62.000	63.000	62.742	0.438	4.570
AOA			63.000	66.000	65.065	0.801	8.441
POA	unrh1	33	34.000	35.000	34.581	0.502	4.789
PSO			34.000	36.000	34.935	0.442	5.865
SBOA			34.000	35.000	34.097	0.296	3.324
AOA			35.000	36.000	35.387	0.487	7.234
POA	uclr10	25	25.000	28.000	25.548	0.995	2.194
PSO			25.000	28.000	25.742	1.032	2.968
SBOA			25.000	27.000	25.774	0.791	3.097
AOA			25.000	28.000	26.774	0.940	7.097
POA	ucyc08	342	360.000	369.000	363.355	2.214	6.245
PSO			361.000	370.000	365.290	2.355	6.811
SBOA			361.000	370.000	365.935	2.109	6.999
AOA			373.000	383.000	377.645	2.163	10.423

**Table 25:** Times per USCP instance (V3-ELIT).

MH	Instance	Min. Time (s)	Max. Time (s)	Avg. Time (s)	Std. Time (s)
POA	u41	9.469	11.469	10.229	0.582
PSO		7.311	9.248	8.256	0.427
SBOA		11.113	17.587	13.139	1.618
AOA		8.474	11.373	9.866	0.747
POA	u51	14.668	16.855	15.504	0.626
PSO		10.375	11.859	11.061	0.391
SBOA		20.429	27.690	23.172	1.681
AOA		14.597	18.943	16.395	1.116
POA	ua1	33.945	38.881	35.881	1.438
PSO		22.276	26.650	24.191	1.111
SBOA		55.203	76.569	66.592	4.502
AOA		31.992	40.530	35.948	2.184
POA	ub1	27.540	36.632	29.822	1.895
PSO		16.121	18.808	17.223	0.733
SBOA		44.258	65.849	53.597	5.436
AOA		24.627	32.387	27.885	2.121
POA	uc1	66.625	76.951	71.331	2.740
PSO		39.475	46.833	42.263	1.989
SBOA		119.232	165.134	138.785	12.173
AOA		66.077	80.966	72.972	3.549
POA	ud1	55.253	90.119	60.648	7.718
PSO		28.540	33.836	30.652	1.351
SBOA		99.773	135.640	116.104	10.127
AOA		47.504	62.987	53.573	3.385
POA	unre1	92.010	162.368	103.626	18.944
PSO		43.630	55.081	47.754	3.193
SBOA		180.083	215.996	196.142	8.632
AOA		71.796	82.418	77.430	2.822
POA	unrf1	107.928	166.008	135.314	25.211
PSO		44.552	55.018	49.186	2.951
SBOA		192.111	228.696	207.948	10.212
AOA		68.968	84.761	78.180	3.936
POA	unrg1	576.927	657.158	611.560	18.445
PSO		1513.644	1930.749	1642.966	90.250
SBOA		1110.865	1545.107	1321.991	122.878
AOA		597.266	666.813	629.759	15.849
POA	unrh1	431.273	695.414	472.877	50.751
PSO		932.749	1204.076	1015.298	63.790
SBOA		779.427	1180.630	1014.579	107.684
AOA		396.032	435.913	415.094	10.154
POA	uclr10	8.139	10.982	9.042	0.698
PSO		3.452	4.358	3.829	0.277
SBOA		10.443	14.621	12.142	1.090
AOA		5.252	7.752	6.088	0.614
POA	ucyc08	496.836	538.904	517.437	9.296
PSO		489.822	521.377	504.006	9.205
SBOA		819.713	1042.744	942.287	46.942
AOA		514.873	601.580	557.387	21.330

**Table 26:** Fitness results per USCP instance using S3-ELIT binarization.

MH	Instance	Opt.	Best	Worst	Avg. Fitness	Std. Fitness	RPD
POA	u41	38	39.000	43.000	41.194	0.910	8.402
PSO			39.000	43.000	41.290	1.039	8.658
SBOA			39.000	42.000	40.419	0.610	6.367
AOA			40.000	43.000	41.161	0.807	8.319
POA	u51	34	36.000	39.000	37.258	0.682	9.579
PSO			36.000	40.000	37.484	0.851	10.243
SBOA			35.000	37.000	36.484	0.615	7.306
AOA			35.000	38.000	37.161	0.723	9.298
POA	ua1	38	41.000	44.000	42.516	0.926	11.886
PSO			41.000	44.000	42.452	0.768	11.717
SBOA			40.000	43.000	41.742	0.670	9.847
AOA			41.000	44.000	42.548	0.910	11.969
POA	ub1	22	23.000	25.000	24.097	0.746	9.533
PSO			22.000	25.000	24.129	0.718	9.678
SBOA			22.000	24.000	23.323	0.590	6.012
AOA			22.000	25.000	23.548	0.614	7.038
POA	uc1	43	46.000	49.000	47.484	0.851	10.427
PSO			45.000	50.000	47.419	1.148	10.277
SBOA			44.000	48.000	46.677	0.963	8.552
AOA			46.000	49.000	47.323	0.857	10.053
POA	ud1	24	25.000	28.000	26.871	0.619	11.962
PSO			26.000	28.000	27.065	0.574	12.769
SBOA			26.000	27.000	26.419	0.493	10.081
AOA			26.000	28.000	26.677	0.532	11.156
POA	unre1	16	17.000	19.000	17.935	0.359	12.097
PSO			17.000	19.000	18.000	0.365	12.500
SBOA			17.000	18.000	17.968	0.177	12.298
AOA			17.000	18.000	17.903	0.296	11.895
POA	unrf1	10	10.000	11.000	10.806	0.402	8.065
PSO			10.000	11.000	10.871	0.341	8.710
SBOA			10.000	11.000	10.774	0.418	7.742
AOA			10.000	11.000	10.871	0.335	8.710
POA	unrg1	60	66.000	70.000	68.452	0.995	14.086
PSO			66.000	71.000	68.581	1.232	13.978
SBOA			66.000	70.000	68.355	0.935	13.925
AOA			67.000	70.000	68.097	0.995	13.495
POA	unrh1	33	36.000	38.000	37.065	0.680	12.315
PSO			36.000	39.000	37.194	0.601	12.706
SBOA			36.000	38.000	37.290	0.579	13.001
AOA			36.000	38.000	36.968	0.400	12.023
POA	uclr10	25	25.000	28.000	26.000	1.238	4.000
PSO			25.000	29.000	26.387	1.308	5.548
SBOA			25.000	29.000	27.355	1.002	9.419
AOA			25.000	28.000	26.226	1.237	4.903
POA	ucyc08	342	364.000	379.000	372.161	3.734	8.809
PSO			367.000	385.000	373.161	3.652	9.111
SBOA			374.000	380.000	377.452	1.738	10.366
AOA			369.000	381.000	375.710	3.082	9.857

**Table 27:** Times per USCP instance (S3-ELIT).

MH	Instance	Min. Time (s)	Max. Time (s)	Avg. Time (s)	Std. Time (s)
POA	u41	12.341	18.091	14.809	1.526
PSO		11.388	12.681	11.913	0.320
SBOA		12.591	16.955	14.754	1.100
AOA		7.139	10.915	8.374	0.868
POA	u51	20.056	28.581	24.051	2.457
PSO		16.730	18.030	17.466	0.355
SBOA		21.448	27.159	23.864	1.788
AOA		12.211	15.963	14.130	1.023
POA	ua1	59.342	81.301	67.354	4.806
PSO		36.855	40.431	38.558	0.861
SBOA		60.293	82.808	69.015	5.740
AOA		24.953	35.270	28.900	2.478
POA	ub1	46.587	62.619	53.183	3.611
PSO		24.916	27.920	26.904	0.680
SBOA		47.177	60.421	53.876	3.160
AOA		20.328	28.262	23.671	1.752
POA	uc1	127.451	166.076	143.564	9.934
PSO		65.113	71.665	68.250	1.753
SBOA		125.734	157.806	145.917	6.370
AOA		44.653	63.253	53.574	4.412
POA	ud1	93.843	118.145	107.535	6.089
PSO		47.684	51.400	49.021	1.039
SBOA		98.385	120.690	111.496	5.507
AOA		39.288	46.458	43.114	1.751
POA	unre1	146.656	180.531	163.097	8.177
PSO		66.084	70.985	68.296	1.107
SBOA		151.839	184.004	171.704	7.065
AOA		57.299	72.999	63.702	4.101
POA	unrf1	167.776	202.200	184.001	9.880
PSO		66.723	70.476	68.193	0.872
SBOA		169.257	201.062	187.750	7.150
AOA		62.314	76.810	68.156	2.874
POA	unrg1	1153.220	1315.802	1247.234	39.331
PSO		3429.720	3754.630	3604.152	82.391
SBOA		1266.156	1335.203	1303.016	17.494
AOA		437.382	487.136	462.750	10.857
POA	unrh1	759.715	823.730	793.478	19.220
PSO		2054.214	2213.738	2120.835	39.675
SBOA		876.685	956.104	907.609	18.836
AOA		183.417	337.811	297.439	37.552
POA	uclr10	9.422	14.422	11.423	1.240
PSO		4.102	6.316	4.836	0.602
SBOA		9.695	13.361	11.160	0.920
AOA		4.232	5.656	4.732	0.325
POA	ucyc08	914.791	1125.109	1026.490	50.527
PSO		734.819	783.830	754.587	11.110
SBOA		965.016	1107.264	1028.288	42.350
AOA		351.558	426.135	382.280	14.015

**Table 28:** Shapiro–Wilk test for KP.

Inst MHS	S1-STD		V1-STD	
	W	*p*-Value	W	*p*-Value
knapPI_1_100_1000_1 POA	1.0000	1.0000	0.7701	0.0000
knapPI_1_100_1000_1 SBOA	1.0000	1.0000	0.6640	0.0000
knapPI_1_100_1000_1 AOA	1.0000	1.0000	0.9459	0.1206
knapPI_1_100_1000_1 PSO	1.0000	1.0000	0.9487	0.1441
knapPI_1_200_1000_1 POA	1.0000	1.0000	0.2698	0.0000
knapPI_1_200_1000_1 SBOA	1.0000	1.0000	0.1756	0.0000
knapPI_1_200_1000_1 AOA	1.0000	1.0000	0.9339	0.0559
knapPI_1_200_1000_1 PSO	1.0000	1.0000	0.9619	0.3275
knapPI_1_500_1000_1 POA	0.6193	0.0000	0.9873	0.9658
knapPI_1_500_1000_1 SBOA	0.5907	0.0000	0.9283	0.0394
knapPI_1_500_1000_1 AOA	0.9689	0.4900	0.9268	0.0360
knapPI_1_500_1000_1 PSO	0.6347	0.0000	0.9514	0.1702
knapPI_1_1000_1000_1 POA	0.9355	0.0620	0.9482	0.1389
knapPI_1_1000_1000_1 SBOA	0.9692	0.4965	0.9193	0.0226
knapPI_1_1000_1000_1 AOA	0.9610	0.3095	0.9679	0.4633
knapPI_1_1000_1000_1 PSO	0.9767	0.7158	0.9709	0.5447
knapPI_1_2000_1000_1 POA	0.9538	0.1985	0.9654	0.4014
knapPI_1_2000_1000_1 SBOA	0.9749	0.6632	0.9631	0.3515
knapPI_1_2000_1000_1 AOA	0.9711	0.5509	0.9691	0.4955
knapPI_1_2000_1000_1 PSO	0.9214	0.0257	0.9748	0.6599
knapPI_1_5000_1000_1 POA	0.9671	0.4434	0.9066	0.0106
knapPI_1_5000_1000_1 SBOA	0.9175	0.0203	0.9009	0.0076
knapPI_1_5000_1000_1 AOA	0.9727	0.5960	0.9552	0.2173
knapPI_1_5000_1000_1 PSO	0.9649	0.3907	0.9841	0.9145
knapPI_1_10000_1000_1 POA	0.9689	0.4896	0.9090	0.0122
knapPI_1_10000_1000_1 SBOA	0.9231	0.0286	0.9393	0.0787
knapPI_1_10000_1000_1 AOA	0.9782	0.7609	0.9763	0.7029
knapPI_1_10000_1000_1 PSO	0.9405	0.0849	0.9726	0.5943

**Table 29:** Shapiro–Wilk test for KP.

Inst MHS	S1-STD		V1-STD	
	W	*p*-Value	W	*p*-Value
knapPI_2_100_1000_1 POA	1.0000	1.0000	0.7522	0.0000
knapPI_2_100_1000_1 SBOA	1.0000	1.0000	0.7644	0.0000
knapPI_2_100_1000_1 AOA	1.0000	1.0000	0.7921	0.0000
knapPI_2_100_1000_1 PSO	1.0000	1.0000	0.8996	0.0070
knapPI_2_200_1000_1 POA	1.0000	1.0000	0.9450	0.1134
knapPI_2_200_1000_1 SBOA	1.0000	1.0000	0.9577	0.2533
knapPI_2_200_1000_1 AOA	1.0000	1.0000	0.8878	0.0036
knapPI_2_200_1000_1 PSO	1.0000	1.0000	0.9448	0.1122
knapPI_2_500_1000_1 POA	0.8056	0.0001	0.6695	0.0000
knapPI_2_500_1000_1 SBOA	0.7315	0.0000	0.7884	0.0000
knapPI_2_500_1000_1 AOA	0.8722	0.0016	0.9634	0.3578
knapPI_2_500_1000_1 PSO	0.8144	0.0001	0.9571	0.2439
knapPI_2_1000_1000_1 POA	0.4492	0.0000	0.9695	0.5063
knapPI_2_1000_1000_1 SBOA	0.8063	0.0001	0.9681	0.4671
knapPI_2_1000_1000_1 AOA	0.9537	0.1971	0.9738	0.6300
knapPI_2_1000_1000_1 PSO	0.8287	0.0002	0.9736	0.6236
knapPI_2_2000_1000_1 POA	0.9728	0.5996	0.9795	0.7983
knapPI_2_2000_1000_1 SBOA	0.9796	0.8008	0.9766	0.7125
knapPI_2_2000_1000_1 AOA	0.9695	0.5052	0.9785	0.7687
knapPI_2_2000_1000_1 PSO	0.9378	0.0717	0.9628	0.3453
knapPI_2_5000_1000_1 POA	0.9702	0.5242	0.9473	0.1312
knapPI_2_5000_1000_1 SBOA	0.9497	0.1529	0.9652	0.3982
knapPI_2_5000_1000_1 AOA	0.9487	0.1433	0.9765	0.7110
knapPI_2_5000_1000_1 PSO	0.9415	0.0910	0.9883	0.9770
knapPI_2_10000_1000_1 POA	0.9867	0.9591	0.9853	0.9364
knapPI_2_10000_1000_1 SBOA	0.9397	0.0810	0.9662	0.4214
knapPI_2_10000_1000_1 AOA	0.9453	0.1154	0.9778	0.7481
knapPI_2_10000_1000_1 PSO	0.9718	0.5694	0.9706	0.5363

**Table 30:** Shapiro–Wilk test for KP.

Inst MHS	S1-STD		V1-STD	
	W	*p*-Value	W	*p*-Value
knapPI_3_100_1000_1 POA	1.0000	1.0000	0.7855	0.0000
knapPI_3_100_1000_1 SBOA	1.0000	1.0000	0.8586	0.0008
knapPI_3_100_1000_1 AOA	0.1756	0.0000	0.7189	0.0000
knapPI_3_100_1000_1 PSO	0.2698	0.0000	0.6609	0.0000
knapPI_3_200_1000_1 POA	1.0000	1.0000	0.8869	0.0034
knapPI_3_200_1000_1 SBOA	1.0000	1.0000	0.9560	0.2284
knapPI_3_200_1000_1 AOA	1.0000	1.0000	0.7591	0.0000
knapPI_3_200_1000_1 PSO	1.0000	1.0000	0.8401	0.0003
knapPI_3_500_1000 POA	0.1756	0.0000	0.9743	0.6431
knapPI_3_500_1000 SBOA	1.0000	1.0000	0.9585	0.2663
knapPI_3_500_1000 AOA	0.3265	0.0000	0.8885	0.0038
knapPI_3_500_1000 PSO	1.0000	1.0000	0.9224	0.0273
knapPI_3_1000_1000_1 POA	0.3694	0.0000	0.9775	0.7390
knapPI_3_1000_1000_1 SBOA	0.4645	0.0000	0.9239	0.0299
knapPI_3_1000_1000_1 AOA	0.7848	0.0000	0.9767	0.7149
knapPI_3_1000_1000_1 PSO	0.3722	0.0000	0.9381	0.0733
knapPI_3_2000_1000_1 POA	0.8671	0.0012	0.9737	0.6261
knapPI_3_2000_1000_1 SBOA	0.8228	0.0001	0.8649	0.0011
knapPI_3_2000_1000_1 AOA	0.8529	0.0006	0.9311	0.0470
knapPI_3_2000_1000_1 PSO	0.8420	0.0003	0.9746	0.6534
knapPI_3_5000_1000_1 POA	0.9412	0.0889	0.9021	0.0082
knapPI_3_5000_1000_1 SBOA	0.9252	0.0325	0.9859	0.9459
knapPI_3_5000_1000_1 AOA	0.8078	0.0001	0.8821	0.0027
knapPI_3_5000_1000_1 PSO	0.9711	0.5505	0.9057	0.0100
knapPI_3_10000_1000_1 POA	0.9411	0.0883	0.9550	0.2147
knapPI_3_10000_1000_1 SBOA	0.9486	0.1430	0.8940	0.0051
knapPI_3_10000_1000_1 AOA	0.9807	0.8322	0.9760	0.6949
knapPI_3_10000_1000_1 PSO	0.9283	0.0395	0.9807	0.8328

**Table 31:** Shapiro–Wilk test for SCP.

Inst MHS	S3-ELIT		V3-ELIT	
	W	*p*-Value	W	*p*-Value
41 POA	0.7062	0.0000	1.0000	1.0000
41 SBOA	0.8503	0.0005	0.4848	0.0000
41 AOA	0.8445	0.0004	0.8828	0.0028
41 PSO	0.7137	0.0000	1.0000	1.0000
51 POA	0.4435	0.0000	0.6791	0.0000
51 SBOA	0.8364	0.0003	0.5224	0.0000
51 AOA	0.7661	0.0000	0.5852	0.0000
51 PSO	0.6366	0.0000	0.5718	0.0000
61 POA	0.7117	0.0000	0.5741	0.0000
61 SBOA	0.6717	0.0000	0.5049	0.0000
61 AOA	0.7984	0.0000	0.6956	0.0000
61 PSO	0.7081	0.0000	0.6067	0.0000
a1 POA	0.7680	0.0000	1.0000	1.0000
a1 SBOA	0.8863	0.0033	0.7251	0.0000
a1 AOA	0.8941	0.0052	0.9568	0.2389
a1 PSO	0.7486	0.0000	0.3402	0.0000
b1 POA	0.7670	0.0000	0.6961	0.0000
b1 SBOA	0.7651	0.0000	0.7819	0.0000
b1 AOA	0.7993	0.0001	0.8453	0.0004
b1 PSO	0.7308	0.0000	0.7719	0.0000
c1 POA	0.9175	0.0202	0.7894	0.0000
c1 SBOA	0.8661	0.0011	0.7288	0.0000
c1 AOA	0.8772	0.0020	0.9427	0.0978
c1 PSO	0.9339	0.0559	0.7994	0.0001
d1 POA	0.8956	0.0056	0.8672	0.0012
d1 SBOA	0.7208	0.0000	0.6068	0.0000
d1 AOA	0.8438	0.0004	0.8707	0.0014
d1 PSO	0.9037	0.0089	0.8004	0.0001
nre1 POA	0.2698	0.0000	1.0000	1.0000
nre1 SBOA	1.0000	1.0000	1.0000	1.0000
nre1 AOA	0.4448	0.0000	1.0000	1.0000
nre1 PSO	0.1756	0.0000	1.0000	1.0000
nrf1 POA	0.4848	0.0000	0.1756	0.0000
nrf1 SBOA	1.0000	1.0000	1.0000	1.0000
nrf1 AOA	0.4448	0.0000	0.1756	0.0000
nrf1 PSO	0.4448	0.0000	0.4448	0.0000
nrg1 POA	0.9087	0.0119	0.9432	0.1011
nrg1 SBOA	0.8876	0.0036	0.8228	0.0001
nrg1 AOA	0.8451	0.0004	0.9254	0.0330
nrg1 PSO	0.9417	0.0920	0.9267	0.0356
nrh1 POA	0.9323	0.0505	0.9034	0.0088
nrh1 SBOA	0.8568	0.0007	0.8196	0.0001
nrh1 AOA	0.8855	0.0032	0.8701	0.0014
nrh1 PSO	0.8754	0.0018	0.8777	0.0021

**Table 32:** Shapiro–Wilk test for USCP.

Inst MHS	S3-ELIT		V3-ELIT	
	W	*p*-Value	W	*p*-Value
U41 POA	0.8697	0.0014	0.8275	0.0002
U41 SBOA	0.7808	0.0000	0.7288	0.0000
U41 AOA	0.8585	0.0008	0.8428	0.0004
U41 PSO	0.9149	0.0173	0.7866	0.0000
U51 POA	0.8163	0.0001	0.8011	0.0001
U51 SBOA	0.7251	0.0000	0.6286	0.0000
U51 AOA	0.7955	0.0000	0.6066	0.0000
U51 PSO	0.8524	0.0006	0.7462	0.0000
ua1 POA	0.8818	0.0026	0.6193	0.0000
ua1 SBOA	0.8163	0.0001	0.3402	0.0000
ua1 AOA	0.8840	0.0029	0.8479	0.0005
ua1 PSO	0.8380	0.0003	0.7128	0.0000
ub1 POA	0.8090	0.0001	0.4848	0.0000
ub1 SBOA	0.7511	0.0000	0.5709	0.0000
ub1 AOA	0.7872	0.0000	0.4866	0.0000
ub1 PSO	0.7904	0.0000	0.6953	0.0000
uc1 POA	0.8680	0.0013	0.7520	0.0000
uc1 SBOA	0.8822	0.0027	0.7397	0.0000
uc1 AOA	0.8735	0.0017	0.7123	0.0000
uc1 PSO	0.9304	0.0449	0.7907	0.0000
ud1 POA	0.7343	0.0000	0.5907	0.0000
ud1 SBOA	0.6286	0.0000	0.1756	0.0000
ud1 AOA	0.7051	0.0000	0.6068	0.0000
ud1 PSO	0.7430	0.0000	0.6068	0.0000
unre1 POA	0.4866	0.0000	0.3402	0.0000
unre1 SBOA	0.1756	0.0000	10.000	10.000
unre1 AOA	0.3402	0.0000	0.3974	0.0000
unre1 PSO	0.5048	0.0000	0.4448	0.0000
unrf1 POA	0.4848	0.0000	0.6347	0.0000
unrf1 SBOA	0.5185	0.0000	0.1756	0.0000
unrf1 AOA	0.3974	0.0000	0.6068	0.0000
unrf1 PSO	0.3974	0.0000	0.6068	0.0000
unrg1 POA	0.9029	0.0085	0.5709	0.0000
unrg1 SBOA	0.8775	0.0021	0.5470	0.0000
unrg1 AOA	0.8504	0.0005	0.8414	0.0003
unrg1 PSO	0.9264	0.0350	0.8277	0.0002
unrh1 POA	0.8011	0.0001	0.6286	0.0000
unrh1 SBOA	0.7470	0.0000	0.3402	0.0000
unrh1 AOA	0.5618	0.0000	0.6193	0.0000
unrh1 PSO	0.7229	0.0000	0.6066	0.0000
uclr10 POA	0.7349	0.0000	0.6045	0.0000
uclr10 SBOA	0.9128	0.0153	0.7767	0.0000
uclr10 AOA	0.7824	0.0000	0.8743	0.0018
uclr10 PSO	0.8162	0.0001	0.7078	0.0000
ucyc08 POA	0.9756	0.6826	0.9507	0.1636
ucyc08 SBOA	0.9099	0.0129	0.9474	0.1326
ucyc08 AOA	0.9772	0.7309	0.9747	0.6559
ucyc08 PSO	0.9392	0.0786	0.9686	0.4805

**Table 33:** Wilcoxon–Mann–Whitney KP.

Instance	S1-STD			V1-STD		
	PSO	AOA	SBOA	PSO	AOA	SBOA
knapPI_1_100_1000_1	1.0	1.0	1.0	0.0001	0.0	0.7402
knapPI_1_200_1000_1	1.0	1.0	1.0	0.0	0.0	0.57
knapPI_1_500_1000_1	0.6154	0.0	0.6044	0.0	0.0	0.9719
knapPI_1_1000_1000_1	0.0049	0.0	0.3527	0.0	0.0	0.5927
knapPI_1_2000_1000_1	0.0324	0.0	0.9215	0.0	0.0	0.7039
knapPI_1_5000_1000_1	0.0202	0.0	0.3074	0.0	0.0	0.132
knapPI_1_10000_1000_1	0.0001	0.0	0.4142	0.0	0.0	0.8327
knapPI_2_100_1000_1	1.0	1.0	1.0	0.0001	0.0	0.9088
knapPI_2_200_1000_1	1.0	1.0	1.0	0.0	0.0	0.1288
knapPI_2_500_1000_1	0.0693	0.0	0.9941	0.0	0.0	0.437
knapPI_2_1000_1000_1	0.1434	0.0	0.2673	0.0	0.0	0.2721
knapPI_2_2000_1000_1	0.0002	0.0	0.8053	0.4992	0.4062	0.1613
knapPI_2_5000_1000_1	0.0113	0.0	0.5263	0.6882	0.2691	0.3278
knapPI_2_10000_1000_1	0.0007	0.0	0.8327	0.151	0.7945	0.2287
knapPI_3_100_1000_1	0.1606	0.3332	1.0	0.0	0.0	0.1217
knapPI_3_200_1000_1	1.0	1.0	1.0	0.0	0.0	0.0015
knapPI_3_500_1000_1	0.3332	0.0	0.3332	0.0	0.0	0.01
knapPI_3_1000_1000_1	0.0374	0.0	0.4594	0.0	0.0	0.181
knapPI_3_2000_1000_1	0.0004	0.0	0.2336	0.0	0.0	0.0329
knapPI_3_5000_1000_1	0.0018	0.0	0.508	0.0209	0.9663	0.683
knapPI_3_10000_1000_1	0.0005	0.0	1.0	0.4513	0.0135	0.6372

**Table 34:** Wilcoxon–Mann–Whitney SCP.

Instance	S3-ELIT			V3-ELIT		
	PSO	AOA	SBOA	PSO	AOA	SBOA
41	0.6241	0.0311	0.0006	1.0	0.0000	0.0110
51	0.6727	0.9571	0.0000	0.5435	0.0000	0.0837
61	0.2390	0.5272	0.0326	0.8220	0.0001	0.4361
a1	0.7561	0.0292	0.0000	0.0815	0.0000	0.0000
b1	0.1767	0.4209	0.2404	0.0770	0.2615	1.0
c1	0.8612	0.0038	0.0000	0.0311	0.0000	0.0000
d1	0.5056	0.2627	0.2952	0.7223	0.1318	0.1911
nre1	0.5700	0.2375	0.1606	1.0	1.0	1.0
nrf1	0.7496	0.7496	0.0110	0.0909	1.0	0.3332
nrg1	0.2835	0.0115	0.0000	0.9143	0.0000	0.6285
nrh1	0.7064	0.9355	0.8414	0.9121	0.0001	0.0073

**Table 35:** Wilcoxon–Mann–Whitney USCP.

Instance	S3-ELIT			V3-ELIT		
	PSO	AOA	SBOA	PSO	AOA	SBOA
u41	0.6649	0.9517	0.0002	0.663	0.1945	0.8392
u51	0.2624	0.7939	0.0001	0.6795	0.9523	0.0368
ua1	0.7572	0.8764	0.0009	0.0004	0.0129	0.0083
ub1	0.8107	0.0048	0.0001	0.865	0.0117	0.0001
uc1	0.7385	0.4749	0.0023	0.0051	0.0000	0.0202
ud1	0.2458	0.1324	0.0017	0.0120	0.0120	0.0031
unre1	0.4906	0.7291	0.6438	0.4597	0.7001	0.0815
unrf1	0.5003	0.5003	0.7646	0.4460	0.4460	0.0001
unrg1	0.7576	0.1348	0.7376	0.000	0.0000	0.7846
unrh1	0.5442	0.4880	0.1884	0.0060	0.0000	0.0001
uclr10	0.2758	0.4750	0.0001	0.4212	0.0000	0.1061
ucyc08	0.3694	0.0003	0.000	0.0020	0.0000	0.0000

## Data Availability

The benchmark instances (KP, SCP, and USCP) used in this study were obtained from the public OR-Library [55] and were used without modification. The source code for the BPOA implementation and experimental scripts is publicly available at https://github.com/ZtockHD123/Solver-For-BPOA, accessed on 23 december 2025.

## Abbreviations

The following abbreviations are used in this manuscript:

POA	Pufferfish Optimization Algorithm
BPOA	Binary Pufferfish Optimization Algorithm
PSO	Particle Swarm Optimization
GWO	Grey Wolf Optimizer
FH	Firefly Algorithm
BSMA	Binary Slime Mould Algorithm
WOA	Whale Optimization Algorithm
KP	Knapsack Problem
SCP	Set Covering Problem
USCP	Unicost Set Covering Problem
STD	Standard
ELIT	Elitist
